# Monitoring the Phenolic and Terpenic Profile of Olives, Olive Oils and By-Products throughout the Production Process

**DOI:** 10.3390/foods13101555

**Published:** 2024-05-16

**Authors:** Lucía López-Salas, Javier Díaz-Moreno, Marco Ciulu, Isabel Borrás-Linares, Rosa Quirantes-Piné, Jesús Lozano-Sánchez

**Affiliations:** 1 Department of Food Science and Nutrition, Faculty of Farmacy, University of Granada, Campus Universitario Cartuja s/n, 18071 Granada, Spain; lucialpz@ugr.es (L.L.-S.); javierdm@correo.ugr.es (J.D.-M.); jesusls@ugr.es (J.L.-S.); 2 Department of Biotechnology, University of Verona, Strada le Grazie 15, Cà Vignal 1, 37134 Verona, Italy; marco.ciulu@univr.it; 3 Department of Analytical Chemistry, Faculty of Sciences, University of Granada, Avda Fuentenueva s/n, 18071 Granada, Spain; rquirantes@ugr.es

**Keywords:** olive oil, olive fruit, olive waste, olive by-products, polyphenols, terpenes, HPLC-MS

## Abstract

Olive oil is a food of great importance in the Mediterranean diet and culture. However, during its production, the olive oil industry generates a large amount of waste by-products that can be an important source of bioactive compounds, such as phenolic compounds and terpenes, revalorizing them in the context of the circular economy. Therefore, it is of great interest to study the distribution and abundance of these bioactive compounds in the different by-products. This research is a screening focused on phytochemical analysis, with particular emphasis on the identification and quantification of the phenolic and terpenic fractions. Both the main products of the olive industry (olives, olive paste and produced oil) and the by-products generated throughout the oil production process (leaf, “alpeorujo”, liquid and solid residues generated during decanting commonly named “borras” and washing water) were analyzed. For this purpose, different optimized extraction procedures were performed for each matrix, followed by high-performance liquid chromatography coupled with electrospray time-of-flight mass spectrometry (HPLC-ESI-TOF/MS) analysis. Although no phenolic alcohols were quantified in the leaf and the presence of secoiridoids was low, this by-product was notable for its flavonoid (720 ± 20 µg/g) and terpene (5000 ± 300 µg/g) contents. “Alpeorujo” presented a complete profile of compounds of interest, being abundant in phenolic alcohols (900 ± 100 µg/g), secoiridoids (4500 ± 500 µg/g) and terpenes (1200 ± 100 µg/g), among others. On the other hand, while the solid residue of the borras was the most abundant in phenolic alcohols (3700 ± 200 µg/g) and secoiridoids (680 ± 20 µg/g), the liquid fraction of this waste was notable for its content of elenolic acid derivatives (1700 ± 100 µg/mL) and phenolic alcohols (3000 ± 300 µg/mL). Furthermore, to our knowledge, this is the first time that the terpene content of this by-product has been monitored, demonstrating that it is an important source of these compounds, especially maslinic acid (120 ± 20 µg/g). Finally, the phytochemical content in wash water was lower than expected, and only elenolic acid derivatives were detected (6 ± 1 µg/mL). The results highlighted the potential of the olive by-products as possible alternative sources of a wide variety of olive bioactive compounds for their revalorization into value-added products.

## 1. Introduction

Olive oil has played a fundamental role in the Mediterranean diet and culture since antiquity, being highly appreciated for its nutritional and organoleptic attributes [1]. Different populations of this region have cultivated olive groves and extracted its oil for thousands of years, making it one of the most consumed oils in the world. Indeed, olive cultivation and the olive oil industry are of great economic and social importance, especially in Mediterranean countries, such as Spain, Italy, Argelia, Turkey, Portugal and Greece [2]. Moreover, olive oil has been associated with numerous beneficial and health-promoting properties because of its high content of monounsaturated and polyunsaturated fatty acids, as well as other minor compounds with important activity, particularly phenolic alcohols and secoiridoid derivatives [3,4,5]. In fact, olive oil polyphenols are associated with reduced morbidity and/or decreased progression of cardiovascular, neurodegenerative and even cancer diseases [6,7,8]. Their mechanism of action links them directly to their antioxidant activity, as they reduce the level of reactive oxygen species in the body [7] and help restore the reserve of antioxidants such as superoxide dismutase or catalase [9]. 

It is important to highlight the main bioactive phenolic families of olives, specifically secoiridoids and phenolic alcohols. Among the first group, one of the major compounds is oleuropein aglycone, which is the main contributor to the bitterness of this product. Its anti-inflammatory and antioxidant effects have been associated with other benefits such as being cardioprotective or neuroprotective, among others [10]. Another important secoiridoid of the olive is oleocanthal, which has been described as the main cause of the potent astringency felt in the throat when consuming olive oil. This compound is related to the potent anti-inflammatory activity exerted by this oil, similar to drugs such as ibuprofen, in addition to its activity as an antioxidant, anticancer, antimicrobial and neuroprotective agent [10,11,12,13,14]. Moreover, oleacein is another important secoiridoid that has been described as an antioxidant, anti-inflammatory and as the main cause of the positive effects on cardiovascular health, as in diseases such as atherosclerosis [10,13,14,15]. Finally, oleuropein, a secoiridoid also present in olives, should not be forgotten. Its main function is antioxidant, since the presence of hydroxyl groups allows it to donate hydrogens that prevent oxidation [16]. Other studied functions of this substance are cardioprotective, neuroprotective and anticancer, among others [17,18,19]. 

On the other hand, within the group of phenolic alcohols, hydroxytyrosol should be highlighted. It is a simple o-diphenol found in fruits, and it is one of the most important and studied compounds in olive oil. Hydroxytyrosol can also be generated by the hydrolysis of oleuropein, which occurs during olive ripening or storage and gives rise to the complexity and variety of flavors present in the oil [20]. Its health benefits lie in its ability to scavenge free radicals and reactive oxygen or nitrogen species, as well as to activate the antioxidant systems of the body [7]. Therefore, this compound has been described as an antioxidant, anti-inflammatory, anti-aging, anticancer and antimicrobial agent [21,22,23]. In addition, its positive effect on the reduction of plasma cholesterol levels has been reported, so its cardioprotective character can also be mentioned [24]. 

In conclusion, terpenes are another group of compounds derived from isoprene that can be found in olive products, to which numerous beneficial properties for the organism are also attributed. In olive products, the most important are triterpenic acids, highlighting oleanolic and maslinic acids [25]. Specifically, oleanolic acid, in addition to its antioxidant activity, has been studied for numerous other health-related functions, such as its antiatherogenic, antihyperlipidemic and hypoglycemic effects [26,27]. On the other hand, it is important to highlight the anti-inflammatory, anticancer, hypoglycemic and neuroprotective activities of maslinic acid, among others [28]. 

The biological interest in these phytochemicals has stimulated research on the phenolic and terpenoid bioactive profiles of olives, olive oils and their by-products. The olive oil industry generates a large number of by-products that are often treated as waste with high environmental and economic costs, which could be used [29]. The olive oil production process includes different stages: harvesting, washing, crushing, malaxing and extraction. The main by-products generated during these stages are leaves and pruning waste (the first by-products generated), solid and semi-solid mill waste derived from olive crushing and olive oil decantation (consisting of skin, stones, pulp and water, generated in large quantities from 581 kg per ton in the two-phase system to 735 kg per ton in the three-phase system) and olive mill wastewater, which includes olive vegetative water and water used during extraction (generated from 200 L/ton in the two-phase system to 1200 L/ton in the three-phase system) [30,31]. 

As the generation of waste is a widespread concern in the food industry, many authors have developed their research with the aim of revalorizing these by-products generated in the context of the circular economy [32,33,34,35,36]. The main objective is to promote their usage for obtaining bioactive ingredients because they are considered rich sources of these mentioned bioactive compounds of high added value. In the case of the olive oil industry, the by-products generated can be an important source of bioactive compounds. However, it is important to consider that the concentration and composition of these bioactive compounds present in the olives, olive oil or by-products will vary depending on certain factors, such as genotype, climate, cultivation and agronomic conditions, or the extraction methods used, among others [37]. 

Therefore, the main objective of this research was to evaluate the phenolic and terpenic compound profiles in olive products and by-products and the changes that occur in these bioactive compound families. For this purpose, the Hojiblanca variety was used because it is one of the olive varieties with the most complete phenolic and terpene profile, being a potential raw material to explore the partition coefficient of these bioactive compounds in (a) the fruit and olive paste; (b) the oil, both obtained in each of the centrifugation and decantation phases and (c) in the by-products, with particular emphasis on polyphenols and terpenes, the latter being less studied in by-products such as “borras”.

## 2. Materials and Methods

### 2.1. Samples

The *Olea europaea* samples analyzed (olive fruit, olive paste, oil, olive leaf—OL, “alpeorujo”—ALP, borras liquid residue—BLR, borras solid residue—BSR and olive fruit washing water—OFWW) were of the Hojiblanca variety collected in February 2023. These samples were provided by the Sociedad Cooperativa Andaluza Olivarera Pontanense in the locality of Puente Genil (Córdoba, Spain). The olives were processed in an industrial plant equipped with a hammer crusher, a horizontal blender, one horizontal and one vertical centrifuge (two-phase system) and a conical decanter.

**Figure 1:**
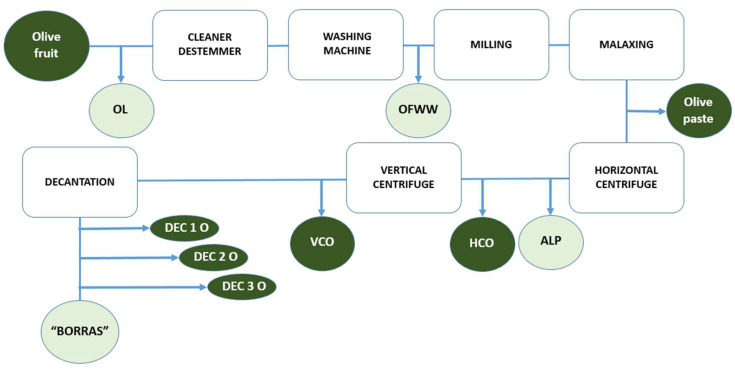
Diagram of the olive oil production process and the generation of the by-products used in the study. OL: olive leaf; OFWW: olive fruit washing water; ALP: “alpeorujo”; HCO: horizontal centrifuge oil; VCO: vertical centrifuge oil; DEC (1,2,3) O: decanted oils 1, 2 and 3.

With regard to the samples analyzed, the first to be mentioned is the olive fruit. To be able to use it and produce olive oil, the first step was to remove the OL and branches, obtaining the first by-product analyzed in this study, as shown in Figure 1. Then, the olive fruit was cleaned and washed in the washing machine, giving rise to the OFWW (second by-product analyzed). After milling and malaxation steps, the olive paste was passed to the horizontal centrifuge (horizontal centrifuge oil is obtained) and then to the vertical centrifuge (vertical centrifuge oil), giving rise to the “alpeorujo” by-product. Finally, the oil was decanted, and during this time, another by-product appeared at the bottom of the decanting tanks, the “borras” (BLR and BSR). Finally, after the decanting stage, 3 samples of decanted oil were collected at different times: decanted oil 1, collected on 10 February; decanted oil 2, on 14 February and decanted oil 3, on 16 February.

The olive fruit, olive paste, ALP and OFWW samples were stored at −20 °C in darkness, while the oil samples and both “borras” residues (in contact with the oil) were stored at room temperature in the dark until immediate sample preparation. Concerning olive leaves, prior to their extraction and analysis, they were submitted to a traditional drying process in a controlled environment for 20 days for optimum conservation and processing. It should be noted that, for each olive product or by-product, a different extraction method was used. The main reason for the use of different extraction methods is the different nature and composition of each sample. After a comprehensive review of the extraction procedures previously described in the literature for this type of matrices, the most appropriate ones were selected for each matrix in terms of selective extraction of the bioactive compounds under study (phenolic and terpenic compounds) avoiding the co-extraction of other components that could interfere in the analysis, making the identification even more complex and causing ion suppression in the mass analyzer.

### 2.2. Chemicals

Methanol and *n*-hexane were purchased from Honeywell (Charlotte, North Carolina, USA), whereas ethanol was acquired from Scharlab (Barcelona, Spain). Double-deionized water was obtained using a Milli-Q system (Millipore, Bedford, MA, USA). Standards of trans-*p*-coumaric acid, hydroxytyrosol, tyrosol, maslinic acid, oleanolic acid and oleuropein were purchased from Sigma-Aldrich (St. Louis, MO, USA), and (+)-pinoresinol was acquired from PhytoLab (Vestenbergsgreuth, Germany). Apigenin and luteolin were purchased from LGC (Teddington, Middlesex, UK), and luteolin-7-O-glucoside, loganin and verbascoside were purchased from Extrasynthese (Lyon, France).

### 2.3. Conventional Extraction Procedure of Polyphenols and Other Polar Compounds from Extra Virgin Olive Oil (EVOO)

The extraction of olive oil phenolic compounds was performed by liquid–liquid extraction, which was adapted with some modifications from López-Huertas, Lozano-Sánchez and Segura-Carretero, 2021 [38]. First, 5 g of olive oil was weighed and dissolved in 10 mL of *n*-hexane, being homogenized for a few seconds in a vortex at 3200 rpm. Then, 10 mL of methanol–water (60:40, *v*/*v*) was used to perform the extraction step, and the mixture was shaken again in the vortex (2250 rpm, 2 min). The oily fraction was separated from the hydroalcoholic fraction by centrifugation at 1150× *g* for 10 min. This process was repeated twice for each sample of oil to extract all the phenolic compounds. The two polar fractions were united and stored at −20 °C until evaporation at the rotary evaporator, at a temperature below 40 °C. Finally, the obtained residue was dissolved in 0.5 mL of methanol–water (50:50, *v*/*v*) for subsequent analysis on the HPLC-MS analytical platform at a concentration of 10 mg/mL.

### 2.4. Conventional Extraction Procedure of Polyphenols, Terpenes and Other Polar Compounds from Olive Fruit and Olive Paste

The extraction of olive fruit and olive paste polyphenols and terpenes was performed as indicated in the research of De Angelis et al., 2015 [39], with some adaptations. For this purpose, 2 g of the homogenized sample was weighed and 4 mL of *n*-hexane was added. The mixture was shaken in a vortex for 5 min at maximum power. The *n*-hexane was separated by centrifugation at 2722× *g*, 4 °C and 10 min and kept under refrigeration for subsequent recovery of the less polar compounds of interest present in this phase. Then, 10 mL of methanol–water (70:30, *v*/*v*) was added and shaken for 10 min in the vortex. The sample was centrifuged again (2722× *g*, 4 °C, 10 min) to separate the hydroalcoholic phase with the phenolic compounds from the sample. The hydroalcoholic phase was stored, and the extraction was repeated with another aliquot of 10 mL of methanol–water (70:30, *v*/*v*). The two polar extracts were collected and joined. Then, they were brought into contact with the *n*-hexane recovered in the first step and shaken in the vortex (30 s). Again, centrifugation was performed to separate both phases (2722× *g*, 4 °C, 5 min). Only the hydroalcoholic phase was collected, which was again subjected to centrifugation to ensure that the suspended solids were separated. The extracts were filtered (0.45 μm filter) and stored at −20 °C until evaporation at the rotary evaporator at 39 °C. After evaporation, the mass of the extract obtained was calculated and reconstituted in a mixture of methanol–water (70:30, *v*/*v*) until a final concentration of 10 mg/mL. 

### 2.5. Conventional Extraction Procedure of Polyphenols, Terpenes and Other Polar Compounds from Olive Leaf (OL)

The protocol established by Ghomari et al., 2019 [40] for the extraction of polyphenols and other polar compounds of OL was followed, with some modifications. Thus, 1 g of dried and crushed olive leaf was macerated with 10 mL of ethanol for 4 h with continuous agitation (300 rpm) at room temperature. Subsequently, the mixture was centrifuged to separate the suspended solids (2722× *g*, 4 °C, 10 min), and the hydroalcoholic phase was collected. The extraction was repeated under the same conditions, but using distilled water. The two extracted solutions were combined and centrifuged (2722× *g*, 4 °C, 10 min), and the supernatant was filtered using 0.45 μm filters. Samples were stored at −20 °C until evaporation at the rotary evaporator at 39 °C. After evaporation, the mass of the extract obtained was calculated and reconstituted in methanol–water (80:20, *v*/*v*) to give a final concentration of 10 mg/mL for HPLC-MS analysis.

On the other hand, terpenes from olive leaf samples were extracted using the protocol reported by Ramírez et al., 2022 [41], with some modifications. For this purpose, 1.25 g of dry leaf powder with 10 mL of methanol–water (1:1, *v*/*v*) was shaken for 30 min at 300 rpm. The mixture was centrifuged (1890× *g*, 20 °C, 5 min), and the extraction was repeated in the same way. The combined extracts were filtered (0.45 μm filter) and stored at −20 °C until evaporation at the rotary evaporator at 39 °C. Once evaporated, the mass of extract obtained was calculated and reconstituted to a concentration of 10 mg/mL using methanol for their analysis. 

### 2.6. Conventional Extraction Procedure of Polyphenols, Terpenes and Other Polar Compounds from Alpeorujo (ALP)

The extraction of olive ALP polyphenols was carried out as indicated in the research of Cecchi et al., 2022 [42] with some modifications. Thus, 2 g of ALP was macerated with 17.5 mL of ethanol–water (80:20, *v*/*v*) for 1 h with continuous agitation (300 rpm) at room temperature. The mixture was centrifuged (2722× *g*, 4 °C, 10 min), and the hydroalcoholic phase was collected. The extraction was repeated under the same conditions, and the mixture was centrifuged again. Then, the extracts were brought into contact with *n*-hexane and shaken in the vortex (5 min). Again, centrifugation was performed to separate both phases. The two extracts were joined and filtered using 0.45 μm filters. Samples were stored at −20 °C until evaporation at the rotary evaporator at 39 °C. After evaporation, the mass of the extract obtained was calculated and reconstituted in ethanol–water (80:20, *v*/*v*) to give a final concentration of 10 mg/mL for HPLC-MS analysis.

In addition, terpenes from ALP samples were extracted using the protocol reported by Fernández-Hernández et al., 2015 [43], with some modifications. For this purpose, 1 g of ALP was macerated with 10 mL of methanol for 1 h at 300 rpm. The mixture was centrifuged (2722× *g*, 4 °C, 6 min). The extracts were filtered (0.45 μm filter) and stored at −20 °C until evaporation at the rotary evaporator at 39 °C. Once evaporated, the mass of extract obtained was calculated and reconstituted to a concentration of 10 mg/mL using methanol for their analysis. 

### 2.7. Conventional Extraction Procedure of Polyphenols, Terpenes and Other Polar Compounds from Borras Solid Residue (BSR)

The extraction of BSR polyphenols and terpenes was performed by solid–liquid extraction, adapted with some modifications from Lozano-Sánchez et al., 2011 [44]. In this procedure, 2.5 g of the solid residue was weighed, and the lipophilic fraction was removed with 10 mL of *n*-hexane after shaking for 1 h (300 rpm). The obtained mixture was centrifuged (2722× *g*, 4 °C, 10 min), and the sample was immersed for 10 min in an ultrasonic bath with 10 mL of methanol–water (75:25, *v*/*v*). After that, the sample was shaken for 30 min and centrifuged under the previous conditions. The hydroalcoholic phase plus the *n*-hexane recovered at the beginning were shaken in the vortex for 30 s. The mixture was centrifuged again (2722× *g*, 4 °C, 5 min), and the extracts were filtered with a 0.45 μm filter, before being taken to evaporation at the rotary evaporator at 39 °C. After evaporation, the mass of the extract obtained was calculated and reconstituted with methanol–water (75:25, *v*/*v*) at a concentration as for previous samples. The final extracts were stored at −20 °C.

### 2.8. Conventional Extraction Procedure of Polyphenols, Terpenes and Other Polar Compounds from Borras Liquid Residue (BLR)

The extraction of BLR polyphenols, terpenes and other polar compounds was performed using the protocol reported by Lozano-Sánchez et al., 2011 [44]. In this extraction method, 8 mL of *n*-hexane was added to 4 mL of BLR and shaken for 1 h (300 rpm). The samples were centrifuged at 2722× *g* for 10 min, and the aqueous fractions were collected and filtered using a 0.45 µm filter. The filtrate was evaporated at the rotary evaporator at the same temperature as the previous samples and reconstituted until obtaining a final concentration of 10 mg/mL. All extracts were stored at −20 °C.

### 2.9. Conventional Extraction Procedure of Polyphenols and Other Polar Compounds from Olive Fruit Wash Water (OFWW)

The extraction of OFWW polyphenols was performed by a liquid–liquid extraction adapted by Lozano-Sánchez et al., 2011 [44]. Therefore, 20 mL of *n*-hexane was added to 10 mL of OFWW and shaken for 1 h at 300 rpm. The samples were centrifuged at 2722× *g* during 10 min, and the aqueous fractions were filtered with a 0.45 µm filter. The filtrate was evaporated at 39 °C. Then, it was reconstituted in 2 mL of water and sonicated for 3–4 min in an ultrasonic bath to ensure that they were completely reconstituted, with a final concentration of 2.8 mg/mL for HPLC-MS analysis. Again, they were filtered with a 0.45 µm filter and stored at −20 °C. 

### 2.10. HPLC–ESI-TOF-MS Analysis

To perform the analytical characterization of the compounds of interest, two different optimized methods were used: one for the characterization of the oil samples and the other for the characterization of the olive fruit and by-products.

The analysis of the oil samples was carried out using a previously validated method [45]. HPLC analyses of the extracts obtained were performed with an RRLC 1200 series liquid chromatograph (Agilent Technologies, Palo Alto, CA, USA) equipped with a vacuum degasser, an autosampler, a binary pump, a column compartment and a diode array detector (DAD). This equipment was coupled with a time-of-flight mass spectrometer, TOF-MS (Bruker Daltonik, Bremen, Germany). The TOF mass analyzer was equipped with an ESI interface (model G1607A, Agilent Technologies, Palo Alto, CA, USA) operating in negative ion mode. The analytical column used for separation was a Zorbax Eclipse Plus C18, 150 mm × 4.6 mm internal diameter, 1.8 µm (Agilent Technologies, Palo Alto, CA, USA). The mobile phase was water with 0.25% acetic acid (solvent A) and methanol (solvent B) eluted according to the following multi-step gradient: 0 min, 5% solvent B; 7 min, 35% solvent B; 13 min, 45% solvent B; 18.5 min, 50% solvent B; 22 min, 60% solvent B; 29 min, 95% solvent B; 36 min, 5% solvent B. The flow rate was 0.5 mL throughout the entire analysis run, the column temperature was maintained at 25 °C and the injection volume was 10 µL. The separated compounds were monitored with DAD (at wavelengths of 240 and 280 nm) and MS analyzer. At this stage, it was necessary to use a flow divider for the coupling with the TOF-MS detector, since the flow rate reaching the TOF detector had to be below 0.25 mL/min to ensure reproducible results and a stable spray in the interface. The MS detection was performed considering a mass range of 50–1000 *m*/*z*. The optimum values for the source parameters were capillary voltage of +4.5 kV; drying gas temperature, 190 °C; drying gas flow, 9 L/min and nebulizer gas pressure, 2.0 bar. The optimum values for the transfer parameters were capillary output voltage, −150 V; skimmer 1 voltage, −50 V; hexapole 1 voltage, −23 V; hexapole RF, 100 Vpp and skimmer 2 voltage, −22.5 V. External calibration of the mass spectrometer was performed using sodium acetate solution (5 mM sodium hydroxide in water/2-propanol 1:1 (*v*/*v*), with 0.2% acetic acid) as a calibrant in high precision calibration (HPC) regression mode. The calibration solution was injected at the beginning of each run, and all spectra were calibrated prior to compound identification.

On the other hand, the analysis of the olive fruit and by-product samples was carried out on the same analytical platform (HPLC-DAD-ESI-TOF-MS) and stationary phase. On the contrary, the mobile phases consisted of water with 0.1% formic acid (A) and acetonitrile (B) using a gradient elution according to the following multi-step gradient: 0 min, 5% B; 2 min, 30% B; 25 min, 95% B; 40 min, 95% B; 45 min, 5% B. Before the next injection, the initial conditions were maintained for 5 min to equilibrate the system. Flow rate, column temperature, injection volume and mass spectrometer parameters were the same as those described above for oil samples analysis. In this case, external calibration of the instrument was carried out in the same way using sodium formate solution as the calibrant solution, prepared as follows: 5 mM sodium hydroxide and 0.2% formic acid in water–isopropanol (1:1, *v*/*v*). 

Lastly, the quantitative analysis of the samples was carried out on the same analytical platform (HPLC-ESI-TOF-MS) according to the chromatographic and mass spectrometric conditions described previously. To carry out the quantification of the analytes identified in all extracts, triplicate injections of each extraction replicate for each type of sample were performed to ensure the reproducibility of both, the extraction process and the analysis. To carry out the quantitation, calibration curves were prepared with 9 commercially available standards; some of them present in the extracts and others with similar structures to other identified compounds in the samples. The stock solutions of each of these standards (oleanolic acid, maslinic acid, apigenin, luteolin, luteolin-7-glucoside, loganin, verbascoside, oleuropein, hydroxytyrosol, tyrosol, pinoresinol and coumaric acid) were prepared in methanol at a concentration of 1000 ppm and stored at −20 °C. Then, solutions of different concentrations of these standards were prepared in the same solvent at concentrations of 0.5, 1, 5, 10, 25, 50, 70 and 100 mg/L. The calibration curves showed good linearity between the different concentration ranges depending on the studied analyte considered. In addition, the limits of detection (LOD) and quantification (LOQ) of each standard with the analytical method for olive fruit and by-products were calculated by direct injection of standard solutions with decreasing amounts of each compound, as the concentration giving peaks for which the signal-to-noise ratio was 3 and 10, respectively. The concentration of the phenolic compounds present in the samples was calculated using the individual area of each compound of the chromatogram and by interpolating in the corresponding calibration curve equation of the commercial standard when available or of a structurally similar one.

The repeatability of the method described for the olive fruit and by-product samples was measured as relative standard deviation % (RSD%) in terms of concentration. A methanolic extract was injected (*n* = 3) on the same day (intraday precision) and 3 times on the 3 consecutive days (interday precision, *n* = 9). Intraday repeatability of the developed method (for all the analytes) was from 0.69 to 5.24%, whereas the interday repeatability was from 1.62 to 8.66%.

For the analysis of the samples, the obtained mass data of the molecular ions were processed using Data Analysis 4.0 and Target Analysis 1.2 software (Bruker Daltonik), whereas the acquisition was performed using HyStar 3.2 software (Bruker Daltonik).

### 2.11. Statistical Analysis 

The extraction of phenolic compounds and terpenes from each olive product and by-product was carried out in triplicate. The resulting data were statistically treated using SPSS v.28 (version 28, IBM^®^ SPSS^®^ Statistics, Armonk, NY, USA). One-way analysis of variance (ANOVA, Tukey’s test) at a 95% confidence level (*p* ≤ 0.05) was performed to point out the differences in quantitative bioactive compound contents found in the olive products and by-product samples with statistical significance.

## 3. Results

### 3.1. Identification of Phenolic Compounds by HPLC–ESI-TOF-MS

#### 3.1.1. Identification of Bioactive Compounds in Olive Fruit, Olive Paste and Olive Oils

**Figure 2:**
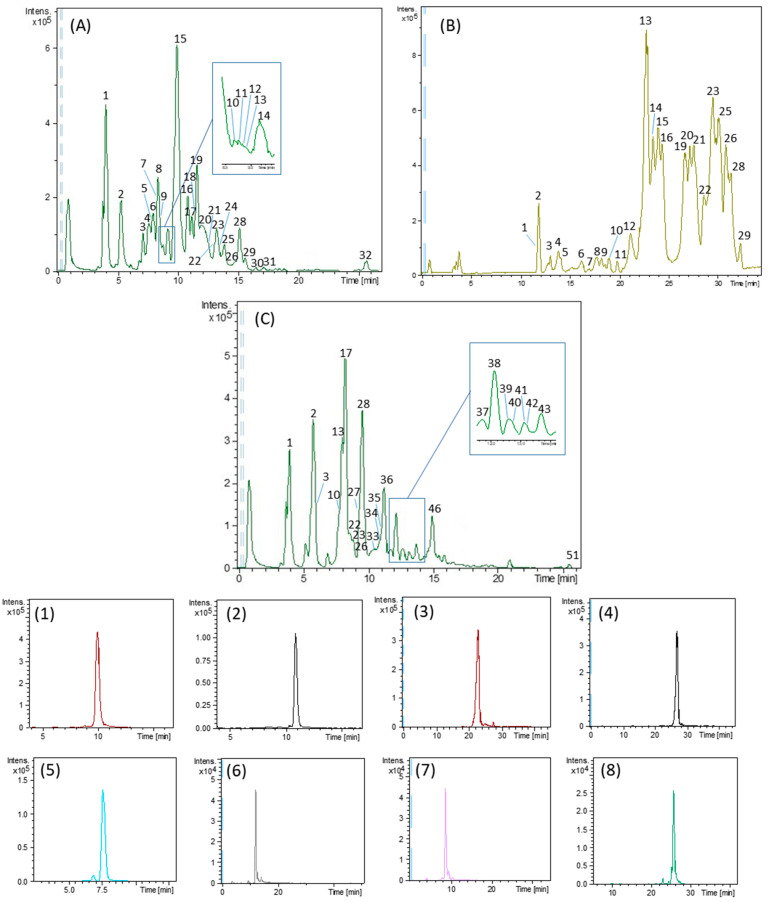
BPC of representative olive fruit (**A**), oil (**B**) and borras solid residues (**C**) extracts and EIC of some phenolic compounds characterized by HPLC-ESI-TOF/MS: (**1**) oleuropein, (**2**) ligstroside, (**3**) oleacein, (**4**) oleocanthal, (**5**) hydroxytyrosol glucoside, (**6**) luteolin, (**7**) verbascoside, (**8**) maslinic acid.

Representative base peak chromatograms (BPCs) of the analyzed extracts and the extracted ion chromatograms (EICs) of some phenolic compounds characterized by HPLC-ESI-TOF/MS are shown in Figure 2. The compounds were characterized thanks to the chemical information provided by the HPLC-ESI-TOF-MS instrument. In this sense, some of the detected compounds were tentatively identified by comparing their retention time and mass spectra with the available commercial standards. All other compounds for which commercial standards were not available were identified by the interpretation of their mass spectra and the molecular formula provided by the DataAnalysis 4.0 software, together with the information previously reported in databases and literature concerning olive composition.

Thus, a total of 32 compounds were putatively identified in the analyzed olive fruit and olive paste extracts, and 29 compounds were characterized in the olive oil extracts, as shown in Table 1 and Table 2. These tables summarized the following information: retention time (RT), experimental and theoretical *m*/*z*, error (ppm), molecular formula and the proposed compound for each chromatographic peak. Most of these tentative compounds have been previously identified in olive products [38,44,46,47,48,49,50,51,52,53,54,55]. 

In the case of olive fruit and olive paste extracts (Table 1), belonging to the group of phenolic alcohols, hydroxytyrosol, peak 8, with *m*/*z* 153 and molecular formula C_8_H_10_O_3_, and its derivatives, oxidized hydroxytyrosol (peak 3) and hydroxytyrosol glucoside (peak 5), were characterized. With regard to the secoiridoid family, oleuropein (peak 15, *m*/*z* 539 and molecular formula C_25_H_32_O_13_) was identified, together with its derivatives hydroxyoleuropein (peak 12) and dihydrooleuropein (peak 14), 6 isomers of oleuropein aglycone (peaks 17, 19, 23, 25, 28 and 29) and decarboxymethyl oleuropein aglycone, also called oleacein (peak 20, *m*/*z* 319 and molecular formula C_17_H_20_O_6_). In addition, ligstroside, peak 16, with *m*/*z* 523 and molecular formula C_25_H_32_O_12_, together with its derivative ligstroside aglycone (5 isomers, peaks 22, 24, 26, 30 and 31) were also found. Oleocanthal (peak 27, *m*/*z* 303 and molecular formula C_17_H_20_O_5_), another ligstroside derivative formed during technological processing of olives such as milling and malaxing, was only detected in olive paste. The identification of numerous isomers of oleuropein and ligstroside aglycone forms has already been described in other research works [38,56]. It has been suggested that possible transformations between these isomers are due to the effect of the tautomeric keto-enolic equilibrium in olive oil on the labile hemiacetal carbon that is exposed after the removal of the glucose [56].

On the other hand, verbascoside, peak 11, with *m*/*z* 623 and molecular formula C_29_H_36_O_15_, has also been previously described as an olives phenolic compound in other research works. This compound belongs to the phenylpropanoid/phenylethanol family. The next family of compounds detected was flavonoids, of which only luteolin (peak 21, *m*/*z* 285 and molecular formula C_15_H_10_O_6_) and its glucosylated derivative (peak 13) were identified. 

Apart from these phenolic families, other compounds belonging to oleoside and elenolic acid derivatives were characterized. In this regard, oleoside (peak 7, *m*/*z* 389 and molecular formula C_16_H_22_O_11_), elenolic acid (peak 18, *m*/*z* 241 and molecular formula C_11_H_14_O_6_) and its derivatives decarboxymethylated elenolic acid (peak 10), 1-β-D-glucopyranosyl acyclodihydroelenolic acid (peak 6) and elenolic acid glucoside (peak 9) were identified. Concerning the identification of terpenes, only loganic acid (peak 4, *m*/*z* 375 and molecular formula C_16_H_24_O_10_) and maslinic acid (peak 32, *m*/*z* 471 and molecular formula C_30_H_48_O_4_) were identified. Finally, other tentatively proposed compounds were quinic acid (peak 1, *m*/*z* 191 and molecular formula C_7_H_12_O_6_) and citric acid (peak 2, *m*/*z* 191 and molecular formula C_6_H_8_O_7_). No compounds belonging to the phenolic acid or lignan families were characterized in these products.

Regarding the oils studied, the different oils obtained during the production process of the final EVOO were analyzed: horizontal centrifuge oil, vertical centrifuge oil and decanted oils 1, 2 and 3. In contrast to olive fruits and paste, phenolic acids were found in their composition, specifically *p*-coumaric acid (peak 10, *m*/*z* 163 and molecular formula C_9_H_8_O_3_). Moreover, the phenolic alcohols characterized in these samples were hydroxytyrosol (peak 2) and two of its derivatives (peak 1, oxidized hydroxytyrosol and peak 11, hydroxytyrosol acetate), as well as tyrosol, peak 5, with *m*/*z* 137 and molecular formula C_8_H_10_O_2_. In addition, the proposed compounds belonging to the secoiridoid group were oleuropein derivatives, such as hydroxy-D-oleuropein aglycone (peak 6, *m*/*z* 335 and molecular formula C_17_H_20_O_7_), 10-hydroxyoleuropein aglycone (peak 7, *m*/*z* 393 and molecular formula C_19_H_22_O_9_), oleuropein aglycone (6 isomers, peaks 14, 15, 16, 22, 23 and 25) and oleacein (peak 13). Furthermore, ligstroside-derived compounds, such as ligstroside aglycone (5 isomers, peaks 20, 21 26, 28 and 29) and decarboxymethyl ligstroside aglycone (also called oleocanthal, peak 19), with *m*/*z* 303 and molecular formula C_17_H_20_O_5_, were also characterized.

Following the lignan group, the tentatively identified compounds were syringaresinol (peak 17, *m*/*z* 417 and molecular formula C_22_H_26_O_8_) and pinoresinol (peak 18, *m*/*z* 357 and molecular formula C_20_H_22_O_6_). Furthermore, in the flavonoid group, luteolin (peak 24, *m*/*z* 285 and molecular formula C_15_H_10_O_6_) and apigenin (peak 27, *m*/*z* 269 and molecular formula C_15_H_10_O_5_) were identified. Moreover, two isomers of elenolic acid (peaks 4 and 12) and decarboxymethylated elenolic acid (peak 3, *m*/*z* 183 and molecular formula C_9_H_12_O_4_) were also characterized. Finally, it should be noted that in olive oil samples, no compound belonging to the terpene group or any other family was identified. Despite all the efforts made for characterization, two compounds detected in the extracts (peaks 8 and 9, both with *m*/*z* 255 and molecular formula C_12_H_16_O_6_) could not be identified and named as unknown (UK 1 and 2).

Comparing olive fruits, paste and oil samples, some notable differences were found. Olives extracts were abundant in glucosylated compounds, while in paste and oils, compounds in their aglycone form stood out. For example, in the olive fruit, within the family of phenolic alcohols, there was the hydroxytyrosol glucoside; luteolin glucoside was characterized in the flavonoids group; 1-β-D-glucopyranosyl cyclodihydroelenolic acid, oleoside and elenolic acid glucoside within the oleoside and elenolic acid derivatives, in addition to verbascoside. On the contrary, luteolin, apigenin, oleuropein and ligustroside were found in their aglycone form in the oil. Moreover, aglycone forms derived from oleuropein were also characterized in oils: as hydroxy-D-oleuropein aglycone, 10-hydroxyoleuropein aglycone, oleacein (decarboxymethyl oleuropein aglycone); and ligustroside derivatives, such as ligustroside aglycone and oleocanthal (decarboxymethyl ligustroside aglycone), belonging to the secoiridoids family. The olive paste presented a more intermediate composition, since glycosylated compounds were identified such as hydroxytyrosol glucoside and luteolin glucoside, but it was also rich in aglycone forms such as hydroxytyrosol and aglycone oleuropein and ligustroside derivatives. 

It is also important to highlight that a total of 16 compounds were identified in common in the samples, olives, paste and oil. These compounds were hydroxytyrosol (phenolic alcohol), luteolin (flavonoid), 6 isomers of oleuropein aglycone, oleacein and 5 isomers of ligustroside aglycone (secoiridoids), elenolic acid and its decarboxymethylated derivative. Although their presence in olive oil is probably due to the hydrolysis that occurs in the sample treatment for technological oil extraction, some of them have been described as olive fruit components in the literature [50,57]. 

#### 3.1.2. Identification of Bioactive Compounds Present in By-Products of the Olive Oil Production Process

A total of 52 compounds were tentatively identified in the characterization performed on the samples of by-products from the olive oil production process (OL, ALP, BSR, BLR and OFWW). Most of these compounds have been previously identified in other studies carried out on these matrices [42,44,46,47,48,49,52,53,54,55,58,59,60]. Table 3 lists the different proposed compounds ordered by retention time (RT), other analytical information as in the previous section and also the by-product or by-products in which each compound was detected indicated in the last column. 

First, belonging to the phenolic alcohol family, hydroxytyrosol (peak 13) and its oxidized derivative (peak 3) were also identified. Tyrosol (peak 22) and 3,4-dihydroxyphenylglycol (peak 2, *m*/*z* 169 and molecular formula C_8_H_10_O_4_) were also detected. With regard to the secoiridoid group, a larger number of compounds were found, namely oleuropein (peak 33, *m*/*z* 539 and molecular formula C_25_H_32_O_13_) and several of its derivatives such as hydroxyloleuropein (peak 21), dihydrooleuropein (peak 25), hydroxyoleuropein aglycone (4 isomers, peaks 27, 34, 37 and 41), hydroxydescarboxymethyl oleuropein aglycone (peak 35), oleuropein aglycone (3 isomers, peaks 42, 43 and 46), oleacein (peak 39) and hydrated oleuropein aglycone (peak 40).

In relation to flavonoids, diosmetin (peak 45, *m*/*z* 299 and molecular formula C_16_H_12_O_6_) and two glucoside isomers (peaks 19 and 32), luteolin (peak 38, *m*/*z* 285 and molecular formula C_15_H_10_O_6_) and its glucoside form (peak 29), and apigenin (peak 44, *m*/*z* 269 and molecular formula C_15_H_10_O_5_) together with its glycosided derivatives apigenin-7-O-glucoside (peak 30) and apigenin-7-O-rutinoside (peak 24) were characterized. Concerning the phenylpropanoid/phenyletanol family, verbascoside (peak 20, *m*/*z* 623 and molecular formula C_29_H_36_O_15_) was identified only in the ALP extracts.

With regard to the oleoside and elenolic acid derivatives group, oleoside (peak 6, with *m*/*z* 389 and molecular formula C_16_H_22_O_11_) was also identified. In addition, numerous compounds derived from elenolic acid were identified. Apart from elenolic acid (peak 36, *m*/*z* 241 and molecular formula C_11_H_14_O_6_), an aldehydic form of decarboxymethyl elenolic acid (peak 26), hydroxyelenolic acid (peak 23) and the decarboxylated form of hydroxyelenolic acid (peak 14) were also detected in these samples, among others. 

Regarding the terpenes group, loganic acid (peak 9), 7-epiloganin (peak 12), lamiol (peak 15), methyl jasmonate (peak 47), maslinic acid (peak 51, *m*/*z* 471 and molecular formula C_30_H_48_O_4_), oleanolic acid (peak 52, *m*/*z* 455 and molecular formula C_30_H_48_O_3_) and 2 derivatives (peaks 48 and 49), as well as hydroxy-oxo-oleanolic acid (peak 50) were characterized. Finally, another compound identified as not belonging to these families in the studied by-products was quinic acid (peak 1), a carboxylic acid with *m*/*z* 191 and molecular formula C_7_H_12_O_6_. 

In summary, the group of compounds derived from oleoside and elenolic acid derivatives is the most abundant group with up to 16 derivatives identified, followed by the secoiridoid family with 12 compounds detected. The rest of the groups present a smaller number of characterized compounds. In addition, no compounds belonging to phenolic acid or lignan families were identified in any of the by-products.

### 3.2. Quantification of Phenolic Compounds by HPLC–ESI-TOF-MS

#### 3.2.1. Quantification of Bioactive Compounds in Olive Fruit, Paste and Olive Oils

In order to quantify the amount of the different polar compounds present in olive fruit, olive paste, olive oils and olive by-products, the individual area of each compound was interpolated in the equation of its corresponding calibration curve based on structure similarity. Thus, for each compound, the calibration curve of its commercial standard was used when possible, or the one that was most structurally similar in cases in which no standard was available. The commercial standards used, together with their calibration ranges, calibration equation obtained, coefficient of determination (R^2^) and LOD and LOQ, for the analytical method for olive fruit and by-products are described in Tables S1 and S2. 

For the group of phenolic acids, the standard employed for the quantification of *p*-coumaric acid was coumaric acid. With regard to the phenolic alcohol family, the hydroxytyrosol standard was used to quantify hydroxytyrosol and its derivatives, while the tyrosol standard was used to quantify tyrosol. Furthermore, 3,4-dihydroxyphenylglycol was quantified with the coumaric acid calibration curve. Regarding lignans, the standard employed was pinoresinol, used in the quantification of syringaresinol and pinoresinol. For the flavonoids group, luteolin was used as a standard for the quantification of luteolin and diosmetin. For the glycosylated derivatives of diosmetin, luteolin and apigenin, the luteolin-7-glucoside standard was used. Furthermore, the apigenin standard was employed for the quantification of apigenin. Lastly, the verbascoside standard was used for its quantification. Concerning the quantification of terpenes, the loganin standard was employed for loganic acid, 7-epiloganin, lamiol and methyl jasmonate. For its part, the maslinic acid standard was used for the quantification of this compound, oleanolic acid and other derivatives, such as dihydroxyoleanolic acid and hydroxy-oxo-oleanolic acid. Moreover, all the compounds belonging to secoiridoids, oleoside and elenolic acid derivatives were quantified with the oleuropein standard. 

After the triplicate analysis of the different samples under study by HPLC-TOF-MS, the concentration of each compound in the different samples was quantified using the standards and calibration lines described above. 

**Figure 3:**
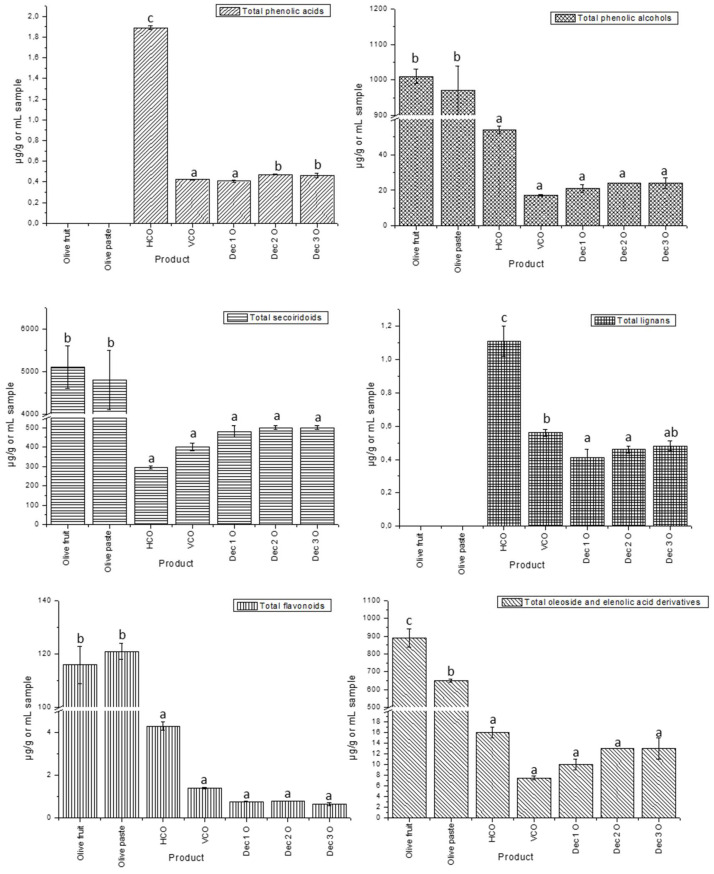
Quantification graphs of the main phenolic compounds in olive fruit, paste and oils. HCO: horizontal centrifuge oil; VCO: vertical centrifuge oil; Dec (1,2,3) O: decanted oils 1, 2 and 3.

Table S3 presents the concentrations of the different compounds that were identified in the olive fruits, paste and oils analyzed, expressed as mean ± standard deviation in µg compound per gram of sample. Figure 3 shows the graphs of the evolution of the concentration of the different families of compounds with processing and time. 

Regarding the phenolic acids, the only compound quantified in the samples, and to which the total concentration was due, was *p*-coumaric acid. The highest amount of this compound was found in the oil from the horizontal centrifuge, with a concentration of 1.89 ± 0.02 µg of phenolic acids per gram of oil. The rest of the oils showed similar concentrations, between 0.406 ± 0.008 and 0.470 ± 0.003 µg/g oil, from decanted oil 1 and decanted oil 2, respectively. However, this compound was not quantified in the present olive fruit or paste samples, despite other research carried out on olives of the Hojiblanca variety detecting the presence of *p*-coumaric acid in the matrix in very low percentages [61]. In other olive varieties such as Arbequina and Manzanilla Cacereña, this compound could not be quantified or was quantified in very low amounts, both in the fruit and in the olive paste [51]. It is possible that it was present in the olive matrix analyzed in this study (since it was detected in the oil samples) but in quantities lower than the detection limit. This small undetectable amount of *p*-coumaric acid could be explained since it is a precursor of verbascoside [50], which was found in large quantities in olive fruit and paste.

In the next group, phenolic alcohols, the olive fruit and the olive paste showed similar and higher concentrations compared to oil samples, with 1010 ± 20 and 970 ± 70 µg of phenolic alcohols per gram. This was followed by the oil from the horizontal centrifuge, which presented 54 ± 2 µg/g oil. The rest of the oil samples presented values similar to each other but lower than those mentioned (between 17.31 ± 0.38 and 24.1 ± 0.1 µg compound/g oil, for oil from the vertical centrifuge and decanted 2, respectively). In this group, in the olive fruit sample, practically, the total concentration was due to the presence of the hydroxytyrosol glucoside, with 980 ± 30 µg/g. A small amount of this compound was quantified in the olive paste (79 ± 8 µg/g). However, hydroxytyrosol glucoside was not found in the oil samples, which reinforces the fact that glycosylated forms are found in olive fruits but not in oils due to their degradation during processing. Indeed, previous research stated that this compound is not expected to be found in olive oil, as hydroxytyrosol glucoside is a very polar compound [62]. Moreover, hydroxytyrosol was found in lower concentration in olive fruit (46 ± 7 µg/g), while the quantified amount of this compound was much higher in the paste samples (630 ± 70 µg/g), due to the sample processing. In fact, other studies have also reported hydroxytyrosol glucoside and hydroxytyrosol as the main phenolic alcohols in olives, while tyrosol was not quantified in this matrix [50]. In this sense, the hydroxytyrosol concentrations found in the oil samples studied are within the normal range according to the literature [63]. Furthermore, tyrosol was present in the decanted oils in similar concentrations within a normal level according to the bibliography [63] and to a certain extent higher in the horizontal centrifuge sample.

Regarding secoiridoids, the olive fruit and the olive paste showed much higher concentrations than the oils, with 5100 ± 500 and 4800 ± 700 µg of compound per gram, respectively. The remaining oils showed a similar concentration, but lower than the fruit (between 400 ± 20 and 500 ± 10 µg/g oil), with the exception of the oil from the vertical centrifuge, which showed the lowest concentration (294 ± 8 µg µg/g oil). This behavior could be explained by the fact that the concentrations of some oleuropein derivatives, such as oleuropein aglycone isomers, were quantified in lower amounts than in the oils obtained in subsequent steps of processing. Thereby, during the mechanical crushing processes of the fruit, the phenolic glycosides initially present in the olive undergo a hydrolysis reaction due to the activation of endogenous enzymes of the olive [14]. As a result, oleuropein and ligstroside are not found in the oil in their glucoside forms other than their aglycone derivatives. However, in the case of the horizontal centrifuge oil, the lower quantified amounts of the oleuropein aglycone isomers could be correlated with the high content of oxidized hydroxytyrosol. Hence, hydroxytyrosol is a derivative of oleuropein that is also obtained by enzymatic hydrolysis, and if the conditions of production and/or storage were not adequate, the hydrolysis of oleuropein into hydroxytyrosol and its subsequent oxidation could have been propitiated. Therefore, the presence of oleuropein in the fruit should be highlighted because it represents almost half of the total content of secoiridoids (2400 ± 300 µg/g olive fruit). These results make sense since oleuropein has been described as the main polyphenol of olive fruits [38]. Another compound to highlight for being found in a high concentration in the fruit would be oleacein, with 940 ± 90 µg/g, while oleocanthal was not found in this matrix. Oleacein is usually present in the oil since it is described in the literature that it is generated in the crushing process [14], which would explain the high content of this compound in the olive paste (3600 ± 720 µg/g). However, it has also been claimed that this compound is formed during the ripening period of the olive fruit, contributing to the bitter taste of the fruit [57]. Therefore, the presence of oleacein in high amounts in the olive fruit could be explained as the result of the sum of the oleacein in the fruit plus the oleacein formed during the treatment of the sample from oleuropein degradation. Other studies on olive fruits already described the presence of oleacein in olive fruit samples in very high amounts as in the case of the present study [57]. 

By contrast, all the compounds in the oils appeared in lower concentrations, with the exception of some isomers of the oleuropein aglycone, oleocanthal and oleacein, which showed higher concentrations. As mentioned above, neither oleuropein nor ligstroside was quantified in the oils, which is typical for this matrix, while their aglycone forms were present instead. As can be seen in Table S3, the most abundant compound determined in almost all the oils derived from the ligstroside of olive fruit was oleocanthal, followed by different isomers of ligstroside aglycone. The same occurred in the case of oleacein, which was quantified in a higher amount than the different isomers of oleuropein aglycone in almost all the analyzed samples with the exception of the horizontal centrifuge oil. As stated above for some oleuropein aglycone isomers, this lower amount of oleacein could be related to the high values found for oxidized hydroxytyrosol in this oil. The quantified amounts of the isomers of the aglycone forms of oleuropein and ligstroside were in agreement with those described in the literature [38]. 

For lignans, the oil from the horizontal centrifuge showed the highest concentration, with 1.11 ± 0.09 µg of lignans per gram of oil. Despite this, the other samples presented close concentration values, ranging from 0.41 ± 0.05 to 0.56 ± 0.02 µg/g. Other research has reported similar amounts of these compounds in oil samples [48]. On the opposite, olive fruit and paste did not present these compounds, probably because they were found in small amounts below the detection limit of the analytical procedure. Only two compounds belonging to this group were quantified in the samples, syringaresinol and pinoresinol. Both showed similar concentrations, except in the oil from the horizontal centrifuge, where syringaresinol was the major contributor to the total sum of lignans.

In the case of flavonoids, olive fruit and paste presented again the higher concentrations of compounds, with 116 ± 7 and 121 ± 3 µg of flavonoids per gram, respectively. Therefore, the oils presented lower and similar values between them (0.65 ± 0.08 and 4.3 ± 0.2 µg/g oil, for decanted oil 3 and the sample from the horizontal centrifuge, respectively). For these polyphenols, it is important to highlight the contribution of the glycoside form of luteolin to the total content of flavonoids in the olive matrix (olive fruit and olive paste). This fact was supported by other researchers, who also found luteolin glucoside as the most abundant flavonoid in olives and paste [50,51]. This compound was not quantified in the oils, so again the occurrence of glycosylated compounds in the olive matrix (olive fruit and olive paste), but not in the oils, is reinforced.

In concerns of the phenylpropanoid/phenylethanol group, verbascoside was only quantified in the olive fruit and paste, with 80 ± 5 and 110 ± 10 µg/g, respectively. Other studies confirmed the high content of this compound in olive fruits and paste [51,57]. On the other hand, it was not possible to quantify these compounds in the oils.

Furthermore, in the group of oleoside and elenolic acid derivatives, olive fruit and paste presented the highest concentration. A total of 890 ± 50 µg/g (fruit) and 650 ± 10 µg/g (paste) were quantified, while the oils showed much lower values ranging from 10 ± 1 to 16 ± 1 µg/g oil. Another study stated that the elenolic acid group was the second most abundant in olives [64]. In the fruit sample, the elenolic acid glycoside represented approximately half of the total concentration of this group of compounds. This elenolic acid derivative has been described as an important compound in the olive matrix [65,66], but again, being a glycoside form, it was not detected in oils. 

With regard to terpenes, they were only quantified in the olive fruit and paste samples, with a concentration of 1200 ± 30 and 1060 ± 20 µg/g, respectively. Therefore, terpenes have not been quantified in oils [38,48,67]. Maslinic acid contributed almost the entire sum, with 1140 ± 50 µg/g olive fruit and 1010 ± 50 µg/g olive paste. 

Therefore, when comparing the qualitative and quantitative characterization carried out on the main olive products, some of the obtained results should be highlighted. Although it was not possible to quantify phenolic acids and lignans in olive fruit and paste, their total phenolic contents and concentrations of the rest of the families were higher with respect to the oils samples. Therefore, the olive fruit was the product with the highest content of phenolic compounds and terpenes. Examples were hydroxytyrosol glucoside (980 ± 30 µg/g), oleuropein (2400 ± 300 µg/g), ligstroside (260 ± 10 µg/g), luteolin glucoside (100 ± 6 µg/g) and maslinic acid (1140 ± 50 µg/g), among others. With a very similar composition and content of phenols and terpenes, olive paste was also a product that stood out. It makes sense since the paste is the same product as the olives, with the difference being the milling and malaxing processes. However, its content of oxidized hydroxytyrosol (absent in the olive fruit) evidenced a certain instability of the product, which is evident since it is the product resulting from the technological processing of the olive fruit.

The results obtained for the oil were also as expected, since oil is a derived product from the olive fruit. These phytochemicals, and especially phenolic compounds, are distributed in various parts of the fruit, such as the skin, pulp and pit. During the oil extraction process, these solids are removed by centrifugation or decanting, resulting in the loss of these substances and consequently a lower concentration of them in the oil compared to the fruit.

It was remarkable that some classes of phenolic compounds were only found in detectable amounts in one matrix or in the other. For example, phenolic acids, represented by *p*-coumaric acid, were only quantified in the oil samples. However, another compound directly related to it, verbascoside, was quantified in large amounts in olive fruit and paste. On the opposite, a compound that was not quantified in these samples, probably because it was below the detection limit, was tyrosol. These facts were also reported in previous research, as mentioned above. The same occurred with lignans, compounds typically found in low amounts in olive oil derived from their presence in the fruit, since they are not products of hydrolysis reactions. The most probable reason is that their content in olive fruit and paste did not exceed the detection limit of the method. On the other hand, as expected, terpenes were not detected in the oil samples, being especially abundant in the olive matrix. These compounds tend to be discarded together with the solid and liquid wastes resulting from the centrifugation of the olive paste, such as “alpeorujo”, as already indicated by other authors [68].

In addition, the fact that glycoside forms of compounds, such as hydroxytyrosol glucoside, luteolin glucoside, oleuropein or ligstroside, among others, predominate in the olive fruit was strongly reinforced. These compounds were not detected in the oil samples studied and were found in smaller quantities in the olive paste. The explanation for this occurrence is that the glycoside forms of the compounds are degraded by enzymes giving rise to the aglycone forms (hydroxytyrosol, luteolin, elenolic acid, oleuropein aglycone isomers, ligstroside aglycone isomers, etc.) during mechanical processing of the olive fruit. Moreover, glycosidic compounds are more water-soluble than their aglycone forms due to the presence of polar functional groups of the sugar moiety. Therefore, a large part of these compounds would be lost together with the aqueous solid and liquid residues resulting from the olive paste centrifugation and decantation steps. Thus, this would explain the marked decrease in the content of phenolic alcohols, secoiridoids, elenolic acid derivatives and flavonoids in oils with respect to the olive fruit and paste contents. 

The enzymatic degradation also explains the presence of oleocanthal in the oils and its absence in the fruit. This compound is the decarboxymethylated form of the ligstroside aglycone, and it is also formed during the crushing and malaxating processes of the olive fruit. Despite the fact that this compound was detected in olive paste, it was not possible to determine its amount (concentration between detection and quantitation limits). One possible explanation could be that oleocanthal had not yet formed in large quantities at this stage. As could be seen in Table S3, the olive fruit and olive paste contents in oleocanthal precursors ligstroside and ligstroside aglycone did not vary greatly. However, it should also be noted that oleacein was detected in olive fruit, paste and oil matrices. Although this compound is also formed during the mechanical processing of the olive fruit, it has been described that its presence in the fruit is due to the enzymes responsible for the fruit ripening process, being obtained from oleuropein [14,57]. Moreover, as expected, numerous aglycone isomers were obtained from oleuropein and ligstroside in the oil samples.

Finally, it is necessary to mention that the oils from the vertical and horizontal centrifugation showed some differences in relation to their composition. For example, the oil from the horizontal centrifuge presented a higher concentration of phenolic alcohols, its main compound oxidized hydroxytyrosol. In addition, other compounds closely related to hydroxytyrosol, such as oleuropein derivatives, were found in lower amounts in this sample than in the rest of the oils, specifically oleuropein aglycone isomers and oleacein. This behavior could be explained by two hypotheses: (a) In the oil from the vertical centrifuge, part of the polar compounds would have separated from the oily phase during the previous horizontal centrifugation phase; (b) the water content in the oil from the vertical centrifuge would be lower (second oil centrifugation), and consequently, the degradation of compounds would be less compared to that can occur in the previous horizontal centrifuge phase.

#### 3.2.2. Quantification of Bioactive Compounds in By-Products of the Olive Oil Production Process

**Figure 4:**
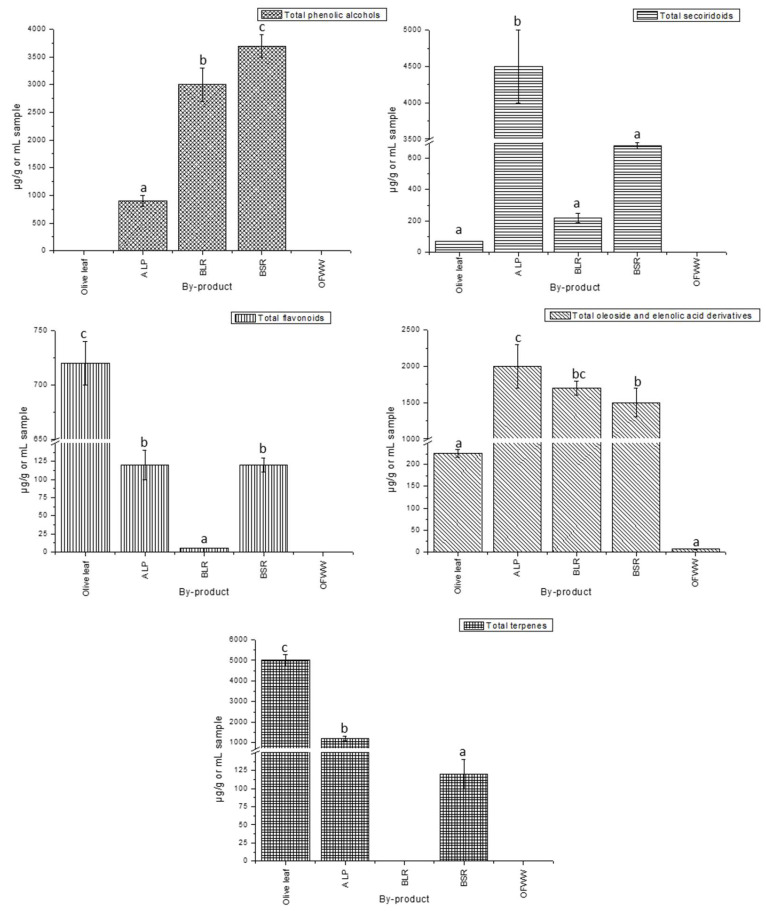
Quantification graphs of phenolic alcohols, secoiridoids, flavonoids, oleoside and elenolic acid derivatives and terpenes in the by-products. ALP: alpeorujo; BLR: borras liquid residue; BSR: borras solid residue; OFWW: olive fruit washing water.

On the other hand, the quantification of the compounds detected in the by-products obtained from the oil production process, specifically OL, ALP, BSR, BLR and OFWW, was also carried out. Table S4 shows the concentrations of the different compounds characterized in these by-products, expressed as mean ± standard deviation in µg compound per gram of sample or mL of sample. Additionally, Figure 4 shows the content of the determined compounds by families in these different by-products.

Regarding the group of phenolic alcohols, it can be observed that only ALP, BSR and BLR showed these kinds of compounds. BSR and BLR samples presented a similar concentration, with 3000 ± 300 µg of phenolic alcohols per mL of BLR, and 3700 ± 200 µg of phenolic alcohols per gram of BSR. It could be observed that the predominant compound was oxidized hydroxytyrosol, contributing to a large part of the total sum of phenolic alcohols. In addition, a special mention should also be made for the contribution of hydroxytyrosol and 3,4-dihydroxyphenylglycol to this total content. These results are in agreement with those obtained for other olive oil storage by-products, in which the presence of phenolic alcohols was higher than in the analyzed oils [48]. On the other hand, the phenolic alcohol content of ALP was also high. Hydroxytyrosol was the most abundant compound in this by-product, which is in agreement with the literature consulted [42]. Neither the OL nor the OFWW showed this group of compounds. In other research carried out in OL, the content of phenolic alcohols was very low or not indicated [52,54], and with respect to OFWW, other authors also confirmed the absence of these compounds in this by-product [58].

For the next group, secoiridoids, the highest concentration appeared in the ALP, with 4500 ± 500 µg/g. The major compound to which more than half of the secoiridoid content was due was oleacein. Other authors also indicated that this compound was the main in the residues of “alpeorujo” [46]. On the other hand, significant amounts of these compounds were also quantified in BSR (680 ± 20 µg/g), which is in agreement with the results obtained in solid wastes from previous research [44]. In addition, the BLR and OL also showed these compounds, but in smaller amounts (220 ± 30 µg/mL BLR and 69 ± 1 µg/g OL). It should be noted that ALP and BSR by-products were the only ones in which it was possible to quantify oleacein, while oleocanthal was not identified in any of the by-products. This means that the oleocanthal generated in the production of the oils does not migrate to these by-products. With respect to oleacein, a considerable amount would be lost in ALP and BSR, compared to the quantities obtained for the analyzed oils (3500 ± 500 µg oleacein/g ALP, 18 ± 2 µg oleacein/g BSR and 51–144 µg oleacein/g oil).

On the other hand, in the case of the OL, it is noteworthy that oleuropein was mainly in its hydroxylated form, exceeding the content of oleuropein itself. Other authors also reported remarkable proportions of different isomers of hydroxyoleuropein in OL [69]. In the case of the present research, no oleuropein aglycone derivatives were detected in OL, whereas in other studies very low amounts were quantified [54]. In addition, oleacein and oleocanthal were also not quantified in the OL, as expected. With regard to OFWW, secoiridoids were not detected as in other research [58]. Within this family, the most abundant compounds that contributed to the total of secoiridoids quantified were one of the isomers of hydroxy oleuropein aglycone in the BLR and BSR, some oleuropein aglycone isomers in the ALP and BSR and hydroxydecarboxymethyl oleuropein aglycone in the ALP.

With regard to flavonoids, the OL showed the highest concentration, with 720 ± 20 µg/g OL. High values of flavonoids in leaves of different olive varieties have also been reported by other studies [70,71]. Thus, the ALP and BSR also presented flavonoids in their composition, but in a much lower concentration (120 ± 20 µg/g ALP and 120 ± 10 µg/g BSR). Regarding BLR, a small amount of these compounds were detected, concretely 4.9 ± 0.3 µg/mL of sample. It is important to mention that luteolin was the only flavonoid from the olives quantified in the BLR and BSR. In other research, luteolin was also the major flavonoid in the oil storage residues, being more numerous in the solid residue than in the liquid by-product [44]. In the same research, the apigenin content was very low, and in our case, undetectable. Regarding ALP, other studies have highlighted the importance of different isomers of the luteolin glucoside in this by-product [53]. Again, the OFWW did not present these compounds, as expected [58]. On the other hand, in the OL, there was a wider variety of compounds quantified, with the highest concentrations found for luteolin glucoside, diosmetine, apigenin-7-O-rutinoside and diosmetine glucoside. 

Concerning the phenylpropanoid/phenyletanol family, verbascoside was only quantified in the ALP, so it could be said that all the verbascoside in the olives is lost in this by-product, since this compound was also not found in the oils. With regard to the oleoside and elenolic acid derivatives, the residues of “borras” and APL presented similar and high concentrations. The BSR showed a concentration level of 1500 ± 200 µg/g; the BLR, 1700 ± 100 µg/mL and the ALP, 2000 ± 300 µg/g. It has already been described in the literature that the elenolic acid derivatives group is very abundant in ALP and olive oil storage by-products [44,48,53,72]. On the contrary, the OFWW showed the lowest concentration of all the studied by-products with 6 ± 1 µg/mL despite it being the most abundant group of compounds quantified for this sample, as in other research on olive mill wastewater [58]. In relation to ALP and the residues of “borras”, which were the samples with the highest concentration of these compounds, hydroxydecarboxymethyl elenolic acid was the one that contributed the most to the total sum of this family, with 600 ± 100 µg/mL BLR, 630 ± 90 µg/g BSR and 460 ± 60 µg/g ALP. Furthermore, elenolic acid (500 ± 80 µg/mL BLR, 260 ± 20 µg/g BSR and 310 ± 50 µg/g ALP) and decarboxymethylated elenolic acid (100 ± 10 µg/mL BLR, 104 ± 4 µg/g BSR and 330 ± 30 µg/g ALP) were also found in high concentration in these samples. Finally, oleoside was obtained in the leaf and the ALP, with 41 ± 3 and 180 ± 10 µg/g. Other studies also reported its importance in these samples [54,69]. 

Therefore, with respect to terpenes, the isomer of dihydroxyoleanolic acid that eluted at a retention time of 18.67 min presented the highest concentration in the OL, with 1490 ± 50 µg/g. Moreover, maslinic acid was found at almost the same concentration, with 1470 ± 90 µg/g of sample. The other isomers of the aforementioned dihydroxyoleanolic acid and hydroxy-oxo-oleanolic acid were also obtained in high concentrations in OL. Other studies also found very high amounts of terpenes in olive leaves of different cultivars [70]. Regarding the ALP, the main terpene was the maslinic acid (1200 ± 100 µg/g ALP), and other research also found this terpene to be one of the main terpene compounds in this by-product [43]. With regard to the other by-products, only BSR had terpenes in its composition, but in a much lower amount (120 ± 20 µg/g). However, although its maslinic acid content is minor, it has been described for the first time in “borras”, and future studies could use this by-product as an alternative source of terpenes in the context of the circular economy.

Therefore, the differences in the composition of phenolic compounds and terpenes of the different by-products studied from the olive oil industry are evident. With respect to the family of phenolic alcohols, they were quite abundant in BLR, BSR and ALP. An important relationship can be established with the phenolic alcohols quantified in olive fruit and paste that did not pass to the oils analyzed. Therefore, a large proportion of phenolic alcohols would be lost in this centrifugation and decanting by-products. However, despite the high content of these compounds, the instability of BLR and BSR was revealed, as the compound quantified in the highest amount in both by-products was oxidized hydroxytyrosol, while this compound was less abundant in ALP. 

In addition, the content of secoiridoids in ALP (4500 ± 500 µg/g) is also noteworthy. These secoiridoids could also be related to those quantified in olive fruit and paste. A large amount of oleuropein (2400 ± 300 µg/g fruit) and its aglycone forms quantified in the fruit and in the paste did not correspond to the aglycone forms quantified in the different oils. Therefore, it was evident that there was a loss of these compounds in the by-products with respect to the oils. Thus, the aglycone forms of oleuropein and its isomers were quantified in significant amounts in ALP but also in other by-products such as “borras”. The main secoiridoid of ALP was oleacein, while only a relevant amount of this compound was found in BRS. Therefore, it can be concluded that olive oil decanting by-products are not particularly rich in this compound of great interest.

On the other hand, it is worth noting the higher concentrations of flavonoid and terpene families found in OL, with important compounds such as luteolin glucoside and apigenin rutinoside, in addition to maslinic and dihydroxyoleanolic acids. Regarding the “borras” residues and ALP, it can be noted that a large amount of these compounds are lost in the form of luteolin and luteolin-glucoside. This would explain the low flavonoid content of the oils, as most of them would be lost in the by-products. In addition, terpenes were only quantified in ALP and RSB, showing a total loss of the fruit terpenes in the by-products generated during the production of olive oil. 

Finally, with respect to oleaoside and elenolic acid derivatives, OL did not stand out for its content unlike ALP (2000 ± 300 µg/g), BLR (1700 ± 100 µg/mL) and BSR (1500 ± 200 µg/g). The content of these by-products is much higher than the content in the fruit (890 ± 50 µg/g fruit), so it could be thought that the secoiridoids present in these by-products are the result of the degradation of other starting substances, thus giving rise to the different elenolic acid derivatives and oxidized hydroxytyrosol.

Therefore, among the olive by-products studied, ALP showed the greatest variety of compounds, with high amounts of important compounds such as phenolic alcohols, secoiridoids, flavonoids and terpenes. Therefore, it could be said that the bioactive profile of ALP is the “most complete”. The residues of the “borras” also presented an interesting phenolic and terpene profile, especially the BSR. Despite its evident in-stability, since the compound quantified in greatest quantity was oxidized hydroxytyrosol, its high content of hydroxytyrosol and oleuropein aglycone makes it a by-product of choice if these compounds need to be recovered. In fact, these compounds, hydroxytyrosol and oleuropein aglycone, are desirable phytochemicals with several applications in the food industry, for example, due to their effect on an increased oxidation resistance in refined olive oils [73]. In addition, they have been shown to be a good source of terpenes, in particular maslinic acid, with numerous beneficial properties [28]. Moreover, if the recovery of flavonoids and terpenes is pursued, another by-product to be selected for its use as a starting material would be OL. 

**Figure 5:**
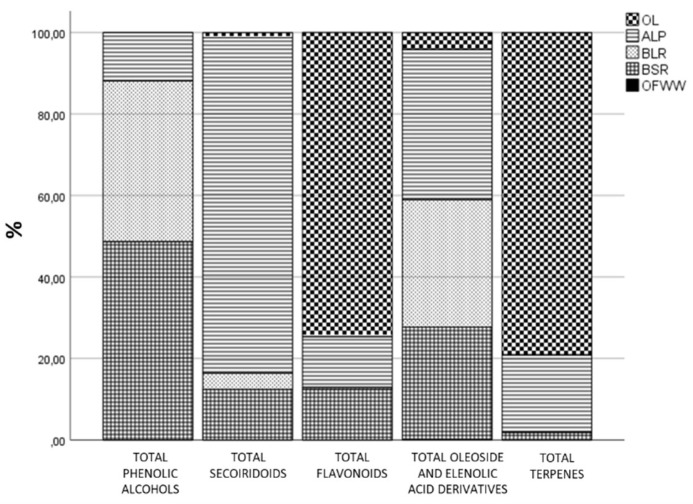
Relative distribution of the target compounds among the different by-products expressed as partition coefficient (%). OL: olive leaf; ALP: alpeorujo; BLR: borras liquid residue; BSR: borras solid residue; OFWW: olive fruit washing water.

In addition, Figure 5 shows the relative distribution of the target compounds among the different by-products expressed as partition coefficient (%) calculated as the percentage of the relative concentration of each family found in a by-product by the sum of the total concentrations detected in all by-products. As can be observed, the olive leaf is the by-product with the highest percentage of flavonoids and terpenes with almost 80% of the total content found in the by-products, making it the by-product of choice for its use as a potential flavonoid and terpene source. In concern to secoiridoids, ALP contains about 80% of the total concentration quantified in the studied by-products, being the preferred by-product as a good source of these compounds. Regarding phenolic alcohols, the by-product that showed about 50% of the total content was BSR, although it should be noted, as mentioned above, that oxidized hydroxytyrosol was the major compound quantified in this by-product. This was also the case for BLR, with about 40% of the total phenolic alcohols found in this by-product. With regard to oleoside and elenolic acid derivatives, these compounds are mainly found in ALP (almost 40%), BLR (around 30%) and BSR (less than 30%).

Finally, by comparing the results obtained for olive products and by-products, it can be observed that higher amounts of phenolic acids (1.89 ± 0.02 µg/g), secoiridoids (5100 ± 500 µg/g) and lignans (1.11 ± 0.09 µg/g) were found in the products compared to the olive by-products. On the other hand, a higher amount of terpenes (5000 ± 300 µg/g), phenolic alcohols (3700 ± 200 µg/g), oleoside and elenolic acid derivatives (2000 ± 300 µg/g) and flavonoids (720 ± 20 µg/g) were found in the olive by-products. These results highlight the potential of these by-products as an enriched source of phytochemicals for their revalorization.

## 4. Conclusions

The olive oil industry generates large quantities of by-products, which are potential sources of compounds with bioactive activities. Among them, phenolic compounds and terpenes stand out and are of great interest for their powerful health benefits. The present research focused on the qualitative and quantitative characterization of the main products of olive cultivation (olives, olive paste and oil) and the major by-products generated throughout the oil production process (leaf, “alpeorujo”, liquid and solid residues of olive “borras” and washing water). 

By comparing the results obtained in the present study, it was possible to conclude that the olive fruit contained a greater variety of phytochemicals belonging to phenolic alcohols, secoiridoids, flavonoids, elenolic acid derivatives and terpenes. As expected, the olive paste presented a similar composition, except for the products resulting from the degradation of the glycosylated phenolic compounds due to the mechanical processing of the olives. On the other hand, among the different analyzed oils, the samples derived from the vertical and horizontal centrifuge showed certain differences in relation to their composition, since the content of phenolic alcohols and secoiridoids were affected and the phenolic alcohols content decreased and the secoiridoid content increased in the vertical centrifuge oil compared to the horizontal centrifuge oil. Regarding the by-products, “alpeorujo” showed a greater variety of compounds with high contents in phenolic alcohols, secoiridoids and other target compounds. The same happened with the BSR, being terpenes (120 ± 20 µg per gram) characterized for the first time in this by-product. However, its stability proved to be limited, since the major compound quantified in this sample was oxidized hydroxytyrosol formed through degradation reactions. Therefore, the highest concentrations of flavonoids and terpenes were found in the olive leaf.

Based on these results, and with a view to future studies oriented to the use of these matrices as a source of bioactive compounds, it should be taken into account which families of compounds are present in higher concentrations in each of the products and/or by-products. Specifically, ALP was the richest by-product in secoiridoids with about 80% of the total, leaf was shown to be a good source of flavonoids and terpenes with almost 80% of their contents, while BLR and BSR were the by-products with higher content in phenolic alcohols. Therefore, this research area about circular economy in the olive oil industry is a field that is generating more and more interest among researchers. However, it needs to be further developed due to the great variability in terms of extraction conditions and the large number of environmental factors that affect the concentration of these bioactive compounds in the olive tree and its derived products and by-products. 

## Figures and Tables

**Table 1:** Identified compounds in the analyzed olive fruit and olive paste extracts.

Peak Number	Proposed Compound	Molecular Formula	RT ^1^ (min)	*m*/*z* (Exp) ^2^	*m*/*z* (Theor) ^3^	Error (ppm)	Product ^4^	References
1	Quinic acid	C_7_H_12_O_6_	4.03	191.0562	191.0561	0.3	OF, OP	[44,48]
2	Citric acid	C_6_H_8_O_7_	5.32	191.0196	191.0203	2.8	OF	[54]
3	Oxydized hydroxytyrosol	C_8_H_8_O_3_	5.90	151.0396	151.0401	3.4	OP	[48]
4	Loganic acid	C_16_H_24_O_10_	7.36	375.1305	375.1297	−2.2	OF, OP	[54]
5	Hydroxytyrosol glucoside	C_14_H_20_O_8_	7.54	315.1091	315.1085	−1.7	OF, OP	[38,49,50]
6	1-β-D-Glucopyranosyl acyclodihydroelenolic acid	C_17_H_28_O_11_	7.69	407.1574	407.1559	−3.8	OF, OP	[53]
7	Oleoside/Secologanoside	C_16_H_22_O_11_	7.97	389.1095	389.1089	−1.4	OF, OP	[49,50]
8	Hydroxytyrosol	C_8_H_10_O_3_	8.14	153.0554	153.0557	2.2	OF, OP	[38,44,48,49,50]
9	Elenolic acid glucoside	C_17_H_24_O_11_	8.36	403.1259	403.1246	−3.3	OF, OP	[49,50]
10	Dialdehydic form of decarboxymethyl elenolic acid	C_9_H_12_O_4_	8.58	183.0659	183.0663	2.0	OF, OP	[48]
11	Verbascoside	C_29_H_36_O_15_	8.63	623.1993	623.1981	−1.9	OF, OP	[49,50,51]
12	Hydroxyoleuropein	C_25_H_32_O_14_	8.68	555.1724	555.1719	0.9	OF, OP	[46,55]
13	Luteolin glucoside	C_21_H_20_O_11_	8.88	447.0944	447.0933	−2.5	OF, OP	[49,50]
14	Dihydrooleuropein	C_25_H_36_O_13_	8.94	543.2075	543.2083	1.5	OF, OP	[47]
15	Oleuropein	C_25_H_32_O_13_	9.88	539.1809	539.1771	−7.1	OF, OP	[49,50,51]
16	Ligstroside	C_25_H_32_O_12_	10.80	523.1824	523.1821	−0.5	OF, OP	[49]
17	Oleuropein aglycone isomer 1	C_19_H_22_O_8_	11.14	377.1250	377.1242	−2.2	OF, OP	[38,44,48,49]
18	Elenolic acid	C_11_H_14_O_6_	11.32	241.0716	241.0718	0.6	OF, OP	[48,49]
19	Oleuropein aglycone isomer 2	C_19_H_22_O_8_	11.55	377.1259	377.1242	−4.6	OF, OP	[38,44,48,49]
20	Oleacein/Decarboxymethyl oleuropein aglycone	C_17_H_20_O_6_	11.97	319.1190	319.1187	0.9	OF, OP	[38,48,49]
21	Luteolin	C_15_H_10_O_6_	12.23	285.0406	285.0405	−0.5	OF, OP	[49,51]
22	Ligstroside aglycone isomer 1	C_19_H_22_O_7_	12.71	361.1295	361.1293	−0.2	OF, OP	[38,48,49]
23	Oleuropein aglycone isomer 3	C_19_H_22_O_8_	13.19	377.1253	377.1242	−2.8	OF, OP	[38,48,49]
24	Ligstroside aglycone isomer 2	C_19_H_22_O_7_	13.26	361.1286	361.1293	1.9	OF, OP	[38,48,49]
25	Oleuropein aglycone isomer 4	C_19_H_22_O_8_	13.86	377.1262	377.1242	−5.3	OF, OP	[38,49]
26	Ligstroside aglycone isomer 3	C_19_H_22_O_7_	14.90	361.1289	361.1293	0.9	OF, OP	[38,48,49]
27	Oleocanthal/Decarboxymethyl ligstroside aglycone	C_17_H_20_O_5_	15.00	303.1231	303.1238	2.4	OP	[38,48,49]
28	Oleuropein aglycone isomer 5	C_19_H_22_O_8_	15.10	377.1257	377.1242	−3.9	OF, OP	[38,49]
29	Oleuropein aglycone isomer 6	C_19_H_22_O_8_	15.57	377.1256	377.1242	−3.8	OF, OP	[38,49]
30	Ligstroside aglycone isomer 4	C_19_H_22_O_7_	15.65	361.1283	361.1293	2.8	OF, OP	[38]
31	Ligstroside aglycone isomer 5	C_19_H_22_O_7_	17.07	361.1284	361.1293	2.3	OF, OP	[38]
32	Maslinic acid	C_30_H_48_O_4_	25.42	471.3491	471.3480	−2.1	OF, OP	[52]

^1^ Retention time; ^2^ Experimental; ^3^ Theoretical; ^4^ OF: olive fruit; OP: olive paste.

**Table 2:** Identified compounds in the analyzed olive oil extracts.

Peak Number	Proposed Compound	Molecular Formula	RT ^1^ (min)	*m*/*z* (Exp) ^2^	*m*/*z* (Theor) ^3^	Error (ppm)	References
1	Oxidized hydroxytyrosol	C_8_H_8_O_3_	11.67	151.0421	151.0401	13.2	[44]
2	Hydroxytyrosol	C_8_H_10_O_3_	11.83	153.0577	153.0557	12.7	[38,44,49]
3	Dialdehydic form of decarboxy methyl elenolic acid	C_9_H_12_O_4_	12.78	183.0669	183.0663	−3.2	[48]
4	Elenolic acid isomer 1	C_11_H_14_O_6_	13.88	241.0713	241.0718	1.8	[48,49]
5	Tyrosol	C_8_H_10_O_2_	14.03	137.0626	137.0608	−18.2	[38,44,48,49]
6	Hydroxy D-oleuropein aglycone	C_17_H_20_O_7_	16.14	335.1130	335.1136	2	[38,48]
7	10-hydroxy oleuropein aglycone	C_19_H_22_O_9_	16.81	393.1181	393.1191	2.6	[48]
8	UK 4 1	C_12_H_16_O_6_	17.68	255.0870	255.0874	1.6	-
9	UK 4 2	C_12_H_16_O_6_	18.30	255.0877	255.0874	1.1	-
10	p-cumaric acid	C_9_H_8_O_3_	19.20	163.0426	163.0401	15.7	[49,50]
11	Hydroxytyrosol acetate	C_10_H_12_O_4_	19.80	195.0665	195.0663	0.9	[38,48,49]
12	Elenolic acid isomer 2	C_11_H_14_O_6_	21.11	241.0721	241.0718	1.2	[48,49]
13	Oleacein/Decarboxymethyl oleuropein aglycone	C_17_H_20_O_6_	22.70	319.1200	319.1187	−4.1	[38,48,49]
14	Oleuropein aglycone isomer 1	C_19_H_22_O_8_	23.43	377.1271	377.1242	−7.8	[38,44,48,49]
15	Oleuropein aglycone isomer 2	C_19_H_22_O_8_	23.95	377.1272	377.1242	−7.8	[38,44,48,49]
16	Oleuropein aglycone isomer 3	C_19_H_22_O_8_	24.37	377.1269	377.1242	−7.2	[38,48,49]
17	Syringaresinol	C_22_H_26_O_8_	25.37	417.1558	417.1555	0.8	[48]
18	Pinoresinol	C_20_H_22_O_6_	26.14	357.1345	357.1344	0.3	[48,49]
19	Oleocanthal/Decarboxymethyl ligstroside aglycone	C_17_H_20_O_5_	26.54	303.1250	303.1238	3.9	[38,48,49]
20	Ligstroside aglycone isomer 1	C_19_H_22_O_7_	27.06	361.1313	361.1293	−5.7	[38,48,49]
21	Ligstroside aglycone isomer 2	C_19_H_22_O_7_	27.40	361.1322	361.1293	−8.2	[38,48,49]
22	Oleuropein aglycone isomer 4	C_19_H_22_O_8_	27.95	377.1258	377.1242	−4.2	[38,49]
23	Oleuropein aglycone isomer 5	C_19_H_22_O_8_	29.35	377.1281	377.1242	−10.3	[38,49]
24	Luteolin	C_15_H_10_O_6_	29.79	285.0407	285.0405	−0.9	[44,48,49]
25	Oleuropein aglycone isomer 6	C_19_H_22_O_7_	29.92	377.1272	377.1242	−8.0	[38,49]
26	Ligstroside aglycone isomer 3	C_19_H_22_O_7_	30.59	361.1323	361.1293	−8.4	[38,48,49]
27	Apigenin	C_15_H_10_O_5_	31.04	269.0451	269.0455	1.5	[44,48,49]
28	Ligstroside aglycone isomer 4	C_19_H_22_O_7_	31.13	361.1312	361.1293	−5.5	[38]
29	Ligstroside aglycone isomer 5	C_19_H_22_O_7_	32.08	361.1302	361.1293	−2.6	[38]

^1^ Retention time; ^2^ Experimental; ^3^ Theoretical; ^4^ UK: unknown compound.

**Table 3:** Identified compounds in the analyzed by-product extracts.

Peak Number	Proposed Compound	Molecular Formula	RT ^1^ (min)	*m*/*z* (Exp) ^2^	*m*/*z* (Theor) ^3^	Error (ppm)	Olive By-Product ^4^	References
1	Quinic acid	C_7_H_12_O_6_	4.03	191.0562	191.0561	0.3	ALP, BLR, BSR	[46]
2	3,4-Dihydroxyphenylglycol	C_8_H_10_O_4_	5.85	169.0509	169.0506	−1.7	BLR, BSR	[49,53]
3	Oxydized hydroxytyrosol	C_8_H_8_O_3_	5.90	151.0396	151.0401	3.4	ALP, BLR, BSR	[44]
4	Hydrated product of the dialdehydic form of decarboxymethyl elenolic acid isomer 1	C_9_H_14_O_5_	7.41	201.0730	201.0768	19.2	OL	[44]
5	Dialdehydic form of decarboxymethyl elenolic acid isomer 1	C_9_H_12_O_4_	7.44	183.0653	183.0663	5.4	BLR	[46,48]
6	Oleoside/Secologanoside	C_16_H_22_O_11_	7.58	389.1103	389.1089	−3.5	ALP, OL	[46,49]
7	1-β-D-Glucopyranosyl acyclodihydroelenolic acid	C_17_H_28_O_11_	7.69	407.1574	407.1559	−3.8	ALP, OL	[53]
8	Hydrated product of the dialdehydic form of decarboxymethyl elenolic acid isomer 2	C_9_H_14_O_5_	7.69	201.0748	201.0768	10.3	OL	[44]
9	Loganic acid	C_16_H_24_O_10_	7.73	375.1288	375.1297	2.3	ALP, OL	[54]
10	Hydrated product of the dialdehydic form of decarboxymethyl elenolic acid isomer 3	C_9_H_14_O_5_	7.93	201.0764	201.0768	2.1	BLR, BSR, OFWW	[44]
11	Hydroxylated product of the dialdehydic form of decarboxymethyl elenolic acid isomer 1	C_9_H_12_O_5_	7.93	199.0598	199.0612	6.8	OL	[44,53]
12	7-Epiloganin	C_17_H_26_O_10_	8.08	389.1430	389.1453	6.0	OL	[55]
13	Hydroxytyrosol	C_8_H_10_O_3_	8.14	153.0554	153.0557	2.2	ALP, BLR, BSR	[42,44,49]
14	Decarboxylated form of hydroxyelenolic acid	C_10_H_14_O_5_	8.23	213.0757	213.0768	5.4	OL, OFWW	[44,48]
15	Lamiol	C_16_H_26_O_10_	8.24	377.1455	377.1453	−0.4	OL	[54]
16	Decarboxymethyl-3,4-dihydroelenolic acid	C_9_H_14_O_4_	8.25	185.0819	185.0815	2.2	ALP, OFWW	[58]
17	Hydroxylated product of the dialdehydic form of decarboxymethyl elenolic acid isomer 2	C_9_H_12_O_5_	8.33	199.0615	199.0612	−1.3	ALP, OL, BLR, BSR, OFWW	[44]
18	Elenolic acid glucoside	C_17_H_24_O_11_	8.36	403.1259	403.1246	−3.3	ALP, OL	[52]
19	Diosmetin glucoside isomer 1	C_20_H_30_O_12_	8.56	461.1650	461.1664	3.1	OL	[54]
20	Verbascoside	C_29_H_36_O_15_	8.63	623.1993	623.1981	−1.9	ALP	[42,49]
21	Hydroxyoleuropein	C_25_H_32_O_14_	8.68	555.1724	555.1719	0.9	OL	[46,55]
22	Tyrosol	C_8_H_10_O_2_	8.73	137.0590	137.0608	13.4	ALP, BLR, BSR	[42,44,49]
23	Hydroxyelenolic acid	C_11_H_14_O_7_	8.78	257.0660	257.0667	2.5	ALP, BLR, BSR	[48]
24	Apigenin-7-O-rutinoside	C_27_H_30_O_14_	8.91	577.1570	577.1563	−1.3	OL	[49]
25	Dihydrooleuropein	C_25_H_36_O_13_	8.94	543.2075	543.2083	1.5	ALP	[47]
26	Aldehydic form of decarboxymethyl elenolic acid	C_10_H_16_O_5_	8.95	215.0910	215.0925	6.8	ALP, BLR, BSR, OFWW	[44,48]
27	Hydroxyoleuropein aglycone isomer 1	C_19_H_22_O_9_	9.38	393.1190	393.1191	0.3	BLR, BSR	[48]
28	Dialdehydic form of decarboxymethyl elenolic acid isomer 2	C_9_H_12_O_4_	9.45	183.0658	183.0663	2.5	ALP, BLR, BSR	[46,48]
29	Luteolin glucoside	C_21_H_20_O_11_	9.45	447.0937	447.0933	−0.9	ALP, OL	[49]
30	Apigenin-7-O-glucoside	C_21_H_20_O_10_	9.48	431.0981	431.0984	0.7	OL	[49]
31	Dialdehydic form of decarboxymethyl elenolic acid isomer 3	C_9_H_12_O_4_	9.50	183.0657	183.0663	3.0	OFWW	[46,48]
32	Diosmetin glucoside isomer 2	C_22_H_22_O_11_	9.62	461.1090	461.1089	−0.2	OL	[54]
33	Oleuropein	C_25_H_32_O_13_	9.88	539.1809	539.1771	−7.1	ALP, BSR, OL	[49]
34	Hydroxyoleuropein aglycone isomer 2	C_19_H_22_O_9_	10.54	393.1203	393.1191	−3.1	BLR, BSR	[48]
35	Hydroxydecarboxymethyl oleuropein aglycone	C_17_H_20_O_7_	10.77	335.1133	335.1136	1.0	ALP, BLR, BSR	[48,53]
36	Elenolic acid	C_11_H_14_O_6_	11.19	241.0716	241.0718	0.6	ALP, BLR, BSR, OL	[48,49]
37	Hydroxyoleuropein aglycone isomer 3	C_19_H_22_O_9_	11.72	393.1181	393.1191	2.6	BSR	[48]
38	Luteolin	C_15_H_10_O_6_	12.09	285.0406	285.0405	−0.5	ALP, BLR, BSR, OL	[42,44,48,49]
39	Oleacein/Decarboxymethyl oleuropein aglycone	C_17_H_20_O_6_	12.26	319.1190	319.1187	0.9	ALP, BSR	[44,48,49]
40	Hydrated oleuropein aglycone	C_19_H_24_O_8_	12.58	379.1397	379.1398	0.4	ALP, BLR, BSR	[44]
41	Hydroxyoleuropein aglycone isomer 4	C_19_H_22_O_9_	12.88	393.1193	393.1191	−0.4	BLR, BSR	[48]
42	Oleuropein aglycone isomer 1	C_19_H_22_O_8_	13.16	377.1250	377.1242	−2.2	ALP, BLR, BSR	[44,49]
43	Oleuropein aglycone isomer 2	C_19_H_22_O_8_	13.66	377.1259	377.1242	−4.6	ALP, BSR	[44,49]
44	Apigenin	C_15_H_10_O_5_	13.85	269.0451	269.0455	1.7	OL	[44,48,49]
45	Diosmetin	C_16_H_12_O_6_	14.12	299.0552	299.0561	3.0	OL	[52]
46	Oleuropein aglycone isomer 3	C_19_H_22_O_8_	14.85	377.1253	377.1242	−2.8	ALP, BSR	[44,49]
47	Methyl jasmonate	C_13_H_20_O_3_	17.34	223.1320	223.1340	8.8	OL	[59]
48	Dihydroxyoleanolic acid isomer 1	C_30_H_48_O_5_	18.67	487.3421	487.3429	1.7	OL	[60]
49	Dihydroxyoleanolic acid isomer 2	C_30_H_48_O_5_	19.92	487.3410	487.3429	4.0	OL	[60]
50	Hydroxy-oxo-oleanolic acid	C_30_H_46_O_4_	25.02	469.3304	469.3323	4.1	OL	[54]
51	Maslinic acid	C_30_H_48_O_4_	25.42	471.3491	471.3480	−2.1	ALP, BSR, OL	[52]
52	Oleanolic acid	C_30_H_48_O_3_	31.68	455.3505	455.3531	5.7	OL	[52]

^1^ Retention time; ^2^ Experimental; ^3^ Theoretical; ^4^ OL: olive leaf; ALP: “alpeorujo”; BLR: borras liquid residue; BSR: borras solid residue; OFWW: olive fruit washing water.

## Data Availability

All the data generated by this research have been included in the article. For any assistance, it is possible to contact the corresponding authors.

## Supplementary Materials

The following supporting information can be downloaded at https://www.mdpi.com/article/10.3390/foods13101555/s1, Table S1: Standards and calibration curves used in the quantification of olive oils compounds; Table S2: Standards and calibration curves used in the quantification of olive fruit, olive paste and by-products compounds; Table S3: Quantification of compounds identified in olive fruit, olive paste and olive oils; Table S4: Quantification of compounds identified in olive oil by-products.
