# Supplementary material for: Monitoring the Phenolic and Terpenic Profile of Olives, Olive Oils and By-Products throughout the Production Process
Source: Foods. 2024 May 16;13(10):1555. doi: 10.3390/foods13101555 (PMC11121151; doi:10.3390/foods13101555)
Supplement: Supplementary file 1 [file foods-13-01555-s001.zip › foods-2922978-supplementary.pdf]

Table S1. Standards and calibration curves used in the quantification of olive oils compounds.

Standard	Calibration Range (mg/L)	Calibration Curve	R^2
**Hydroxytyrosol**	0.5 – 100	y = 132539x + 114801	0.99
**Tyrosol**	0.5 – 100	y = 31019x + 23718	0.99
***p*-Coumaric acid**	0.5 – 10	y = 309212x - 109517	0.99
**Oleuropein**	0.5 – 50	y = 327798x + 513007	0.98
**Pinoresinol**	0.5 – 75	y = 155867x + 610554	0.97
**Luteolin**	0.5 – 75	y = 124152x + 343037	0.99
**Apigenin**	0.5 – 10	y = 428877x - 14275	0.98

Table S2. Standards and calibration curves used in the quantification of olive fruit, olive paste and by-products compounds.

Standard	Calibration Range (mg/L)	Calibration Curve	R^2	LOD (mg/L)	LOQ (mg/L)
**Hydroxytyrosol**	0.1 – 100	y = 45928x + 101086	0.99	$2.8 \cdot 10^{-4} \pm 4 \cdot 10^{-5}$	$9.3 \cdot 10^{-4} \pm 1.4 \cdot 10^{-4}$
***p*- Coumaric acid**	0.1 – 50	y = 114386x + 113145	0.99	$1.7 \cdot 10^{-4} \pm 3 \cdot 10^{-5}$	$5.3 \cdot 10^{-4} \pm 9 \cdot 10^{-5}$
**Oleuropein**	0.1 – 100	y = 277645x + 91705	0.99	$8.8 \cdot 10^{-5} \pm 1.1 \cdot 10^{-5}$	$2.9 \cdot 10^{-4} \pm 4 \cdot 10^{-5}$
**Pinoresinol**	0.1 – 100	y = 28193x - 37545	0.99	$6.3 \cdot 10^{-4} \pm 1.0 \cdot 10^{-4}$	$2.7 \cdot 10^{-3} \pm 5 \cdot 10^{-4}$
**Luteolin**	0.1 – 25	y = 239496x + 164132	0.99	$9.3 \cdot 10^{-4} \pm 1.6 \cdot 10^{-4}$	$3.1 \cdot 10^{-3} \pm 7 \cdot 10^{-4}$
**Apigenin**	0.1 – 25	y = 355194x + 197303	0.98	$8.1 \cdot 10^{-5} \pm 1.4 \cdot 10^{-5}$	$2.7 \cdot 10^{-4} \pm 5 \cdot 10^{-5}$
**Loganin**	0.1 – 25	y = 206516x + 120954	0.98	$9.2 \cdot 10^{-5} \pm 1.1 \cdot 10^{-5}$	$3.1 \cdot 10^{-4} \pm 4 \cdot 10^{-5}$
**Verbascoside**	0.1 – 25	y = 199549x - 27184	0.99	$2.6 \cdot 10^{-4} \pm 4 \cdot 10^{-5}$	$8.8 \cdot 10^{-4} \pm 1.3 \cdot 10^{-4}$
**Luteolin-7-glucoside**	0.1 – 25	y = 132767x + 56279	0.99	$2.7 \cdot 10^{-4} \pm 5 \cdot 10^{-5}$	$9.1 \cdot 10^{-4} \pm 1.7 \cdot 10^{-4}$
**Maslinic acid**	0.1 – 70	y = 12107x + 27158	0.98	$4.3 \cdot 10^{-4} \pm 6 \cdot 10^{-5}$	$1.4 \cdot 10^{-3} \pm 2 \cdot 10^{-4}$

LOD: limits of detection
LOQ: limits of quantification

Table S3. Quantification of compounds identified in olive fruit, olive paste and olive oils.

COMPOUNDS	Olive fruit (µg compound/g fruit)	Olive paste (µg compound/g paste)	Olive oil				
			Horizontal centrifuge oil (µg compound/g oil)	Vertical centrifuge oil (µg compound/g oil)	Decanted oil 1 (µg compound/g oil)	Decanted oil 2 (µg compound/g oil)	Decanted oil 3 (µg compound/g oil)
**Phenolic acids**							
**Total phenolic acids**	**-**	**-**	**1.89 ± 0.02** [c]	**0.423 ± 0.004** [a]	**0.406 ± 0.008** [a]	**0.470 ± 0.003** [b]	**0.46 ± 0.02** [b]
p-cumaric acid	ND	ND	1.89 ± 0.02 [c]	0.423 ± 0.004 [a]	0.406 ± 0.008 [a]	0.470 ± 0.003 [b]	0.46 ± 0.02 [b]
**Phenolic alcohols**							
**Total phenolic alcohols**	**1010 ± 20** [b]	**970 ± 70** [b]	**54 ± 2** [a]	**17.31 ± 0.38** [a]	**21 ± 2** [a]	**24.1 ± 0.1** [a]	**24 ± 3** [a]
Hydroxytyrosol	46 ± 7 [a]	630 ± 70 [b]	1.92 ± 0.08 [a]	7.6 ± 0.3 [a]	8.8 ± 0.9 [a]	10.6 ± 0.4 [a]	10 ± 2 [a]
Oxidized hydroxytyrosol	ND	220 ± 20 [b]	24 ± 1 [a]	2.41 ± 0.07 [a]	3.1 ± 0.2 [a]	4.2 ± 0.1 [a]	4.4 ± 0.8 [a]
Hydroxytyrosol glucoside	980 ± 30 [b]	79 ± 8 [a]	ND	ND	ND	ND	ND
Hydroxytyrosol acetate	ND	ND	1.3 ± 0.2 [a]	2.15 ± 0.05 [b]	2.7 ± 0.4 [c]	2.8 ± 0.2 [c]	2.8 ± 0.3 [c]
Tyrosol	ND	ND	26.6 ± 0.5 [c]	5.2 ± 0.3 [a]	6.0 ± 0.6 [ab]	6.6 ± 0.2 [b]	6.6 ± 0.5 [b]
**Secoiridoids**							
**Total secoiridoids**	**5100 ± 500** [b]	**4800 ± 700** [b]	**294 ± 8** [a]	**400 ± 20** [a]	**480 ± 30** [a]	**500 ± 10** [a]	**500 ± 10** [a]
Oleuropein	2400 ± 300 [b]	310 ± 20 [a]	ND	ND	ND	ND	ND

Hydroxyoleuropein	80 ± 8 [b]	11.8 ± 0.6 [a]	ND	ND	ND	ND	ND
Dihydrooleuropein	41 ± 5 [b]	19 ± 3 [a]	ND	ND	ND	ND	ND
Hydroxy D-oleuropein aglycone	ND	ND	0.54 ± 0.02 [a]	1.34 ± 0.03 [b]	3.0 ± 0.1 [c]	5.2 ± 0.1 [e]	4.4 ± 0.2 [d]
10-hydroxy oleuropein aglycone	ND	ND	0.259 ± 0.005 [a]	0.59 ± 0.02 [d]	0.21 ± 0.04 [a]	0.64 ± 0.07 [e]	0.47 ± 0.07 [c]
Oleuropein aglycone isomer 1	160 ± 10 [b]	30 ± 6 [ab]	8.4 ± 0.1 [a]	42 ± 5 [a]	42 ± 8 [a]	47 ± 5 [a]	46 ± 5 [a]
Oleuropein aglycone isomer 2	400 ± 30 [b]	22 ± 4 [a]	8.2 ± 0.5 [a]	20 ± 1 [a]	20 ± 2 [a]	20 ± 1 [a]	20 ± 1 [a]
Oleuropein aglycone isomer 3	210 ± 6 [c]	12 ± 2 [a]	5.8 ± 0.6 [a]	19.1 ± 0.8 [b]	19 ± 2 [b]	21 ± 1 [b]	19 ± 2 [b]
Oleuropein aglycone isomer 4	120 ± 10 [c]	53 ± 10 [b]	3.3 ± 0.5 [a]	5.2 ± 0.4 [a]	5.1 ± 0.5 [a]	6.2 ± 0.3 [a]	5.7 ± 0.4 [a]
Oleuropein aglycone isomer 5	160 ± 30 [c]	123 ± 30 [b]	95 ± 9 [ab]	74 ± 4 [a]	89 ± 8 [a]	93 ± 6 [ab]	89 ± 4 [a]
Oleuropein aglycone isomer 6	40 ± 5 [a]	480 ± 40 [b]	41 ± 3 [a]	43 ± 7 [a]	55 ± 6 [a]	62 ± 7 [a]	66 ± 6 [a]
Oleacein / Decarboxymethyl oleuropein aglycone	940 ± 90 [a]	3600 ± 720 [b]	51 ± 1 [a]	91 ± 2 [a]	147 ± 5 [a]	146 ± 5 [a]	144 ± 4 [a]
Ligstroside	260 ± 10 [b]	220 ± 20 [a]	ND	ND	ND	ND	ND
Ligstroside aglycone isomer 1	22 ± 4 [d]	8.0 ± 0.2 [a]	9 ± 1 [ab]	16.6 ± 0.9 [c]	13.2 ± 0.4 [bc]	14 ± 1 [c]	13.0 ± 0.6 [bc]
Ligstroside aglycone isomer 2	29 ± 5 [c]	57 ± 7 [d]	15 ± 2 [a]	24 ± 1 [bc]	21 ± 1 [ab]	24 ± 1 [bc]	22.3 ± 0.7 [abc]
Ligstroside aglycone isomer 3	18 ± 2 [bc]	7 ± 2 [a]	17 ± 2 [b]	24 ± 1 [d]	21 ± 2 [bc]	22 ± 11 [cd]	22 ± 21 [cd]

Ligstroside aglycone isomer 4	15 ± 2 [b]	8 ± 1 [a]	14 ± 2 [b]	19 ± 1 [cd]	20 ± 1 [cd]	19.0 ± 0.9 [c]	22 ± 1 [d]
Ligstroside aglycone isomer 5	7 ± 1 [b]	47 ± 5 [c]	1.95 ± 0.07 [a]	3.19 ± 0.07 [ab]	3.0 ± 0.3 [ab]	3.1 ± 0.2 [ab]	3.5 ± 0.2 [ab]
Oleocanthal / Decarboxymethyl ligstroside aglycon	ND	NQ	23.2 ± 0.9 [b]	19.58 ± 0.39 [a]	23 ± 1 [b]	24 ± 1 [bc]	25 ± 1 [c]
				**Lignans**			
**Total lignans**	**-**	**-**	**1.11 ± 0.09 [c]**	**0.56 ± 0.02 [b]**	**0.41 ± 0.05 [a]**	**0.46 ± 0.02 [a]**	**0.48 ± 0.03 [ab]**
Syringaresinol	ND	ND	0.74 ± 0.03 [c]	0.37 ± 0.02 [ab]	0.26 ± 0.04 [a]	0.29 ± 0.01 [ab]	0.32 ± 0.03 [ab]
Pinoresinol	ND	ND	0.37 ± 0.04 [b]	0.18 ± 0.02 [a]	0.15 ± 0.01 [a]	0.17 ± 0.02 [a]	0.160 ± 0.008 [a]
				**Flavonoids**			
**Total flavonoids**	**116 ± 7 [b]**	**121 ± 3 [b]**	**4.3 ± 0.2 [a]**	**1.40 ± 0.05 [a]**	**0.77 ± 0.02 [a]**	**0.801 ± 0.006 [a]**	**0.65 ± 0.08 [a]**
Luteolin	17 ± 2 [c]	24 ± 3 [d]	3.8 ± 0.3 [b]	1.1 ± 0.1 [a]	0.56 ± 0.03 [a]	0.60 ± 0.09 [a]	0.48 ± 0.08 [a]
Luteolin glucoside	100 ± 6 [a]	97 ± 6 [a]	ND	ND	ND	ND	ND
Apigenin	ND	ND	0.51 ± 0.04 [c]	0.28 ± 0.02 [b]	0.21 ± 0.02 [a]	0.206 ± 0.006 [a]	0.178 ± 0.005 [a]
				**Phenylpropanoid/phenylethanol**			
**Tota/ phenyletanol**	**80 ± 5 [a]**	**110 ± 10 [b]**	**ND**	**ND**	**ND**	**ND**	**ND**
Verbascoside	80 ± 5 [a]	110 ± 10 [b]	ND	ND	ND	ND	ND
				**Oleoside and elenolic acid derivatives**			
**Total oleoside and elenolic acid derivatives**	**890 ± 50 [c]**	**650 ± 10 [b]**	**16 ± 1 [a]**	**7.5 ± 0.3 [a]**	**10 ± 1 [a]**	**13.00 ± 0.04 [a]**	**13 ± 2 [a]**
Oleoside	220 ± 10 [b]	130 ± 20 [a]	ND	ND	ND	ND	ND

Elenolic acid isomer 1	70 ± 10 [b]	280 ± 30 [c]	0.70 ± 0.02 [a]	3.3 ± 0.2 [a]	4.2 ± 0.4 [a]	5.0 ± 0.3 [a]	5.2 ± 0.6 [a]
Elenolic acid isomer 2	ND	ND	7.6 ± 0.5 [c]	3.6 ± 0.1 [a]	4.4 ± 0.4 [a]	5.6 ± 0.4 [b]	5.9 ± 0.7 [b]
Dialdehydic form of decarbox-ymethyl elenolic acid	109 ± 7 [c]	80 ± 10 [b]	7.4 ± 0.4 [a]	0.55 ± 0.03 [a]	1.2 ± 0.2 [a]	2.4 ± 0.2 [a]	2.1 ± 0.3 [a]
1-β-D-Glucopyranosyl acyclodihydroeleno-lic acid	140 ± 10 [b]	80 ± 10 [a]	ND	ND	ND	ND	ND
Elenolic acid glucoside	350 ± 20 [b]	100 ± 10 [a]	ND	ND	ND	ND	ND
**Terpenes**							
**Total terpenes**	**1200 ± 30** [b]	**1060 ± 20** [a]	**-**	**-**	**-**	**-**	**-**
Loganic acid	62 ± 5 [b]	50 ± 3 [a]	ND	ND	ND	ND	ND
Maslinic acid	1140 ± 50 [b]	1010 ± 50 [a]	ND	ND	ND	ND	ND

ND non-detected

NQ non-quantified

Different letters mean significant differences ($p < 0.05$) between samples for every variable, with increasing letters indicating increasing values.

Table S4. Quantification of compounds identified in olive oil by-products.

COMPOUNDS	Leaf (µg compound/g leaf)	"Alpeorujo" (µg compound/g alpeorujo)	"Borras" liquid residues (µg compound/mL BLR)	"Borras" solid residues (µg compound/g BSR)	Olive fruit washing water (µg compound/mL OFWW)
			**Phenolic alcohols**		
**Total phenolic alcohols**	**-**	**900 ± 100 [a]**	**3000 ± 300 [b]**	**3700 ± 200 [c]**	**-**
Hydroxytyrosol	ND	540 ± 90 [a]	700 ± 100 [a]	1200 ± 100 [b]	ND
Oxidized hydroxytyrosol	ND	170 ± 30 [a]	1300 ± 100 [b]	1500 ± 100 [b]	ND
Tyrosol	ND	100 ± 20 [a]	150 ± 20 [b]	123 ± 4 [ab]	ND
3,4-Dihydroxyphenylglycol	ND	ND	830 ± 80 [a]	830 ± 40 [a]	ND
			**Secoiridoids**		
**Total secoiridoids**	**69 ± 1 [a]**	**4500 ± 500 [b]**	**220 ± 30 [a]**	**680 ± 20 [a]**	**-**
Oleuropein	24 ± 2 [b]	70 ± 10 [c]	ND	7.6 ± 0.4 [a]	ND
Hydroxyoleuropein	46 ± 4	ND	ND	ND	ND
Dihydrooleuropein	ND	36 ± 5	ND	ND	ND
Hydroxyoleuropein aglycone isomer 1	ND	ND	27 ± 5 [a]	58 ± 1 [b]	ND
Hydroxyoleuropein aglycone isomer 2	ND	ND	89 ± 9 [a]	107 ± 1 [b]	ND
Hydroxyoleuropein aglycone isomer 3	ND	ND	22 ± 4 [a]	96 ± 1 [b]	ND
Hydroxyoleuropein aglycone isomer 4	ND	ND	ND	39.3 ± 0.9	ND

Hydroxydecarboxymethyloleuropein aglycone	ND	190 ± 10 [b]	26 ± 4 [a]	49 ± 2 [a]	ND
Oleuropein aglycone isomer 1	ND	400 ± 70 [b]	36 ± 5 [a]	37 ± 4 [a]	ND
Oleuropein aglycone isomer 2	ND	130 ± 20 [b]	ND	53 ± 4 [a]	ND
Oleuropein aglycone isomer 3	ND	36 ± 7 [a]	ND	160 ± 7 [b]	ND
Oleacein / Decarboxymethyl oleuropein aglycone	ND	3500 ± 500 [b]	ND	18 ± 2 [a]	ND
Hydrated oleuropein aglycone	ND	40 ± 5 [b]	21 ± 3 [a]	55 ± 3 [b]	ND
**Flavonoids**					
**Total flavonoids**	**720 ± 20 [c]**	**120 ± 20 [b]**	**4.9 ± 0.3 [a]**	**120 ± 10 [b]**	**-**
Diosmetin	113 ± 8	ND	ND	ND	ND
Diosmetin glucoside isomer 1	98 ± 6	ND	ND	ND	ND
Diosmetin glucoside isomer 2	49 ± 2	ND	ND	ND	ND
Luteolin	50 ± 3 [c]	24 ± 4 [b]	4.9 ± 0.3 [a]	120 ± 10 [d]	ND
Luteolin glucoside	260 ± 10 [b]	90 ± 10 [a]	ND	ND	ND
Apigenin	37 ± 3	ND	ND	ND	ND
Apigenin-7-O-glucoside	21 ± 2	ND	ND	ND	ND
Apigenin-7-O-rutinoside	100 ± 4	ND	ND	ND	ND
**Phenylpropanoid/phenyletanol**					
**Total phenylpropanoid/ phenyletanol**	**-**	120 ± 10	**-**	**-**	**-**
Verbascoside	ND	120 ± 10	ND	ND	ND
**Oleoside and elenolic acid derivatives**					
**Total oleoside and elenolic acid derivatives**	**226 ± 9 [a]**	**2000 ± 300 [c]**	**1700 ± 100 [bc]**	**1500 ± 200 [b]**	**6 ± 1 [a]**

Oleoside	41 ± 3 [a]	180 ± 10 [b]	ND	ND	ND
Elenolic acid	18 ± 2 [a]	310 ± 50 [b]	500 ± 80 [c]	260 ± 20 [b]	ND
Hydrated product of the dialdehydic form of decarboxymethyl elenolic acid isomer 1	7 ± 1	ND	ND	ND	ND
Hydrated product of the dialdehydic form of decarboxymethyl elenolic acid isomer 2	8.3 ± 0.9	ND	ND	ND	ND
Hydrated product of the dialdehydic form of decarboxymethyl elenolic acid isomer 3	ND	ND	150 ± 10 [b]	170 ± 10 [b]	0.8 ± 0.1 [a]
Dialdehydic form of decarboxymethyl elenolic acid isomer 1	ND	ND	71 ± 7	ND	ND
Dialdehydic form of decarboxymethyl elenolic acid isomer 2	6.7 ± 0.2 [a]	330 ± 30 [c]	100 ± 10 [b]	104 ± 4 [b]	ND
Dialdehydic form of decarboxymethyl elenolic acid isomer 3	ND	ND	ND	ND	2.7 ± 0.5
1-β-D-Glucopyranosyl acyclodihydroelenolic acid	83 ± 3 [a]	150 ± 30 [b]	ND	ND	ND
Hydroxylated product of the dialdehydic form of decarboxymethyl elenolic acid isomer 1	15 ± 1	ND	ND	ND	ND
Hydroxylated product of the dialdehydic form of decarboxymethyl elenolic acid isomer 2	7.9 ± 0.8 [a]	460 ± 60 [b]	600 ± 100 [c]	630 ± 90 [c]	0.38 ± 0.07 [a]
Hydroxyelenolic acid	ND	90 ± 20 [a]	240 ± 40 [b]	260 ± 20 [b]	ND
Decarboxylated form of hydroxyelenolic acid	6.8 ± 0.8 [b]	ND	ND	ND	0.9 ± 0.2 [a]

Decarboxymethyl-3,4-dihydroelenolic acid	ND	270 ± 20 [b]	ND	ND	0.37 ± 0.04 [a]
Elenolic acid glucoside	38 ± 2 [a]	130 ± 20 [b]	ND	ND	ND
Aldehydic form of decarboxymethyl elenolic acid	ND	12 ± 2 [b]	43 ± 5 [c]	51 ± 5 [c]	0.24 ± 0.03 [a]
**Terpenes**					
**Total terpenes**	**5000 ± 300** [c]	**1200 ± 100** [b]	**-**	**120 ± 20** [a]	**-**
Loganic acid	16 ± 1 [a]	17 ± 3 [a]	ND	ND	ND
7-Epiloganin	35 ± 2	ND	ND	ND	ND
Lamiol	32 ± 2	ND	ND	ND	ND
Methyl jasmonate	3.9 ± 0.4	ND	ND	ND	ND
Maslinic acid	1470 ± 90 [b]	1200 ± 100 [b]	ND	120 ± 20 [a]	ND
Oleanolic acid	42 ± 7	ND	ND	ND	ND
Dihydroxyoleanolic acid isomer 1	1490 ± 50	ND	ND	ND	ND
Dihydroxyoleanolic acid isomer 2	1070 ± 80	ND	ND	ND	ND
Hydroxy-oxo-oleanoic acid	830 ± 80	ND	ND	ND	ND

ND non-detected

Different letters mean significant differences ($p < 0.05$) between samples for every variable, with increasing letters indicating increasing values.
